# *C. elegans* Agrin Is Expressed in Pharynx, IL1 Neurons and Distal Tip Cells and Does Not Genetically Interact with Genes Involved in Synaptogenesis or Muscle Function

**DOI:** 10.1371/journal.pone.0000731

**Published:** 2007-08-15

**Authors:** Ana Hrus, Gordon Lau, Harald Hutter, Susanne Schenk, Jacqueline Ferralli, Marianne Brown-Luedi, Ruth Chiquet-Ehrismann, Stefano Canevascini

**Affiliations:** 1 Friedrich Miescher Institute for Biomedical Research, Novartis Research Foundation, Basel, Switzerland; 2 Department of Biological Sciences, Simon Fraser University, Burnaby, British Columbia, Canada; Centre de Regulacio Genomica, Spain

## Abstract

Agrin is a basement membrane protein crucial for development and maintenance of the neuromuscular junction in vertebrates. The *C. elegans* genome harbors a putative agrin gene *agr-1*. We have cloned the corresponding cDNA to determine the primary structure of the protein and expressed its recombinant fragments to raise specific antibodies. The domain organization of AGR-1 is very similar to the vertebrate orthologues. *C. elegans* agrin contains a signal sequence for secretion, seven follistatin domains, three EGF-like repeats and two laminin G domains. AGR-1 loss of function mutants did not exhibit any overt phenotypes and did not acquire resistance to the acetylcholine receptor agonist levamisole. Furthermore, crossing them with various mutants for components of the dystrophin-glycoprotein complex with impaired muscle function did not lead to an aggravation of the phenotypes. Promoter-GFP translational fusion as well as immunostaining of worms revealed expression of agrin in buccal epithelium and the protein deposition in the basal lamina of the pharynx. Furthermore, dorsal and ventral IL1 head neurons and distal tip cells of the gonad arms are sources of agrin production, but no expression was detectable in body muscles or in the motoneurons innervating them. Recombinant worm AGR-1 fragment is able to cluster vertebrate dystroglycan in cultured cells, implying a conservation of this interaction, but since neither of these proteins is expressed in muscle of *C. elegans*, this interaction may be required in different tissues. The connections between muscle cells and the basement membrane, as well as neuromuscular junctions, are structurally distinct between vertebrates and nematodes.

## Introduction

Agrin is a large proteoglycan with a prominent function at the developing neuromuscular junction (NMJ) where it plays a pivotal role in the formation and maintenance of the acetylcholine receptor (AChR) clusters. Agrin was discovered more than two decades ago through the observation that trophic factors from the basal lamina extract of electric ray (*Torpedo californica*) were able to induce AChRs clustering on muscles *in vitro* [1]. The protein was subsequently purified from the extract of the synapse rich *Torpedo* electric organ and, based on the observed aggregating activity, was named “agrin”, coming from Greek “ageirein” which means “to assemble” [2]. Further studies revealed that agrin is synthesized by motor neurons that release it into the synaptic cleft where it stably integrates into the synaptic basal lamina (BL), a specialized thin layer of the extracellular matrix (ECM) [3]–[5]. Based on these findings, McMahan proposed the ‘agrin hypothesis’ which states that agrin is a nerve-derived synaptic organizing molecule [6] (reviewed in [7], [8]).

Agrin has been cloned from several vertebrate species including rat [9], chick [10], [11], marine ray (*Torpedo californica*) [12] and man [13]. All described agrin gene orthologues encode a large protein of more than 2000 amino acids with an approximate molecular weight of 225 kDa. Additional O-linked glycosylation by heparan and chondroitin sulphate glycosaminoglycan chains, together with N-linked carbohydrates, raise the molecular weight up to 400–600 kDa [14], [15] (reviewed in [8]). The domain architecture of agrin is characterized by several repeated structural motifs which share homology with follistatin (Kazal-type protease inhibitors), laminin epidermal growth factor (EGF) and laminin globular (lamG) domains. In addition, the protein contains a SEA module (common between sea urchin sperm protein, enterokinase and agrin) flanked by serine/threonine (S/T)-rich regions [9], [10]. Differential transcription of the first exon results in a longer form which is secreted and binds to the basal lamina via its laminin-binding N-terminal agrin (NtA) domain [11], [16], [17] and a shorter isoform which lacks the NtA domain and remains in the membrane as a type II transmembrane protein [18], [19]. Additional alternative splicing, in a tissue-specific manner at two conserved sites, termed A and B in chicken or y and z in rat, gives rise to isoforms with significantly different activities in clustering AChRs [20]–[22]. Isoforms expressed by motoneurons, which contain inserts at the B/z splice site, are active in AChR clustering, whereas agrin expressed by muscle lacks the inserts and does not cluster AChRs.

Despite of numerous studies available, the mechanism of agrin action has not been completely resolved yet. Muscle specific kinase (MuSK) is a transmembrane receptor tyrosine kinase necessary for agrin-induced AChR clustering without direct interaction with agrin. The missing link in this signaling pathway is a hypothetical protein termed MASC (myotube-associated specificity component) able to mediate the interaction between agrin and MuSK [23], [24]. Genetic analysis of agrin and MuSK deficient mice support the common function in AChR clustering [24], [25]. Both mutants die at birth due to breathing failure. Agrin loss of function mutants and mice lacking only z^+^ agrin exons have a significantly reduced number, size, and density of AChRs clusters on the muscle, even though some postsynaptic differentiation is present [24], [26]. In MuSK mutant mice, NMJ synapses and the related specializations can be found neither on the postsynaptic membrane nor in the basal lamina [25]. Signaling downstream of MuSK is still largely unclear. Several reports demonstrated MuSK-related activation of different proteins, leading to AChR clustering. Among them were dishevelled (Dvl), a protein involved in planar cell polarity signaling [27], a cytoplasmic protein Dok-7 [28], and protein casein kinase 2 (CK2) [29], essential for NMJ synaptogenesis *in vitro* and *in vivo*.

In addition to the NMJ, many non-neuronal tissues such as muscle, heart and kidney, express an agrin isoform without inserts at the B/z site [20], [30]. *Alpha*-dystroglycan (α-DG) binds to this alternative splice variant in different tissues with strong affinity, through a carbohydrate-dependent mechanism [31], [32]. In vertebrates, muscle dystroglycan is a central component of a large dystrophin-glycoprotein complex (DGC) connecting the ECM with the intracellular cytoskeleton (reviewed in [33], [34]). Genetic studies on animal models have shown that mutations in many components of the DGC independently lead to the outcome of muscular dystrophies (reviewed in [35]–[37]). Agrin binding to α-DG might contribute to the connection between the ECM and the cytoskeleton thus improving tissue integrity [38]. The interaction between agrin and α-DG is functionally conserved in the formation of the immunological synapse between antigen presenting cells (APCs) and T-cells [39], [40].

The nematode *C. elegans* is a useful model organism with many experimental advantages, e.g. short generation time, easy maintenance, transparent body and simple but specialized organs which make it a powerful tool for genetic analysis [41], [42]. *C. elegans* harbors a gastrointestinal tract, a reproductive system, epithelial, neural, muscle, excretory cells, and even innate immunity pathways [43]. In addition, most of the molecular mechanisms underlying major physiological processes are highly conserved when compared to vertebrates [42]. Therefore, the experimental data obtained from the worm proved to be highly informative and applicable in elucidating many analogous mechanisms in mammals (for a review, see [44]).

NMJs in *C. elegans* have some distinct morphological features when compared to the vertebrate counterparts. Instead of having motoneurons which grow axons towards the muscles they innervate, muscles in *C. elegans* make specialized cell projections called muscle arms, which extend from the muscle bundles to reach the proximal nerve cord [45], [46]. At the sites of contact, the muscle arms make *en passant* synapses to the motor axons that run along the anterioposterior axis. Depending on the type of the neurotransmitter, the NMJ synapses can be excitatory (cholinergic) or inhibitory (GABAergic). Genetic screens for synaptogenesis mutants have identified key players in NMJ formation and structure. Animals carrying mutations in synaptic components often exhibit uncoordinated movements (*unc*), egg-laying defects (*egl*), defecation defects or paralysis. Pharmacological assays with nematocidal drugs, such as the cholinergic agonist levamisole or the acetylcholine esterase inhibitor aldicarb, have been extensively used in screening for mutants that are resistant to these drugs [41]. The genes for several postsynaptic AChR subunits were identified on the basis of the resistance to levamisole, e.g. *unc-29*, *unc-38*, *unc-63*, *lev-1* [41], [47]. Neuromuscular junctions in *C. elegans* are highly dynamic structures. Several proteins have been identified as crucial factors for normal NMJ development. One of them is a transmembrane protein LEV-10. The mutant was identified as weakly resistant to levamisole due to significantly reduced postsynaptic density of AChRs [48]. Interestingly, the LEV-10 extracellular protein domain alone is sufficient to rescue the *lev-10* mutant phenotype, suggesting a novel AChR clustering mechanism.

In vertebrates one of the key factors involved in AChR clustering is the receptor tyrosine kinase MuSK. The gene with the highest similarity to MuSK in *C. elegans* is an orphan receptor KIN-8 (CAM-1) [49], [50]. In addition to the impairment in cell polarity and neuron migration, the *kin-8*/*cam-1* mutants are uncoordinated and have mislocalized AChR subunit ACR-16 [51]. Therefore, KIN-8/CAM-1 in *C. elegans* might be a protein with a role similar to MuSK in vertebrates. Several other synaptic ECM proteins have been implicated in the NMJ formation in the worm, namely collagen XVIII (CLE-1) and nidogen (NID-1) [52]. Single mutants in each of the genes exhibit reduced numbers of the enlarged and diffuse postsynaptic receptor clusters.

Different genetic approaches have been taken to investigate the functions of the vertebrate gene homologues identified in the *C. elegans* genome (reverse genetics) or to identify the previously unknown genes which, if mutated in the worm, result in interesting phenotypes (forward genetics). In reverse genetic approaches, the goal is to learn more about a particular gene of interest and address its mechanisms of action in *C. elegans* [53]. Since in *C. elegans* and *C. briggsae*, two closely related nematode worm species, putative agrin orthologues have been identified on the basis of genomic sequence analysis [54], we decided to take a reverse genetics approach to clone the *C. elegans* agrin cDNA, characterize the protein, and describe its expression pattern. We found expression of agrin in four head neurons, in the distal tip cell of the gonad, and in epithelial cells of the pharynx. We could not detect any agrin in muscle or at NMJs and genetic analysis of agrin mutants did not provide any evidence for a major function of agrin in AChR clustering or muscle function in the worm. However, the known binding of agrin to α-DG in vertebrates seems to be conserved in *C. elegans*, pointing to an ancestral role of this interaction.

## Results

### *C. elegans* expresses an agrin-like gene *agr-1*

**Figure 1:**
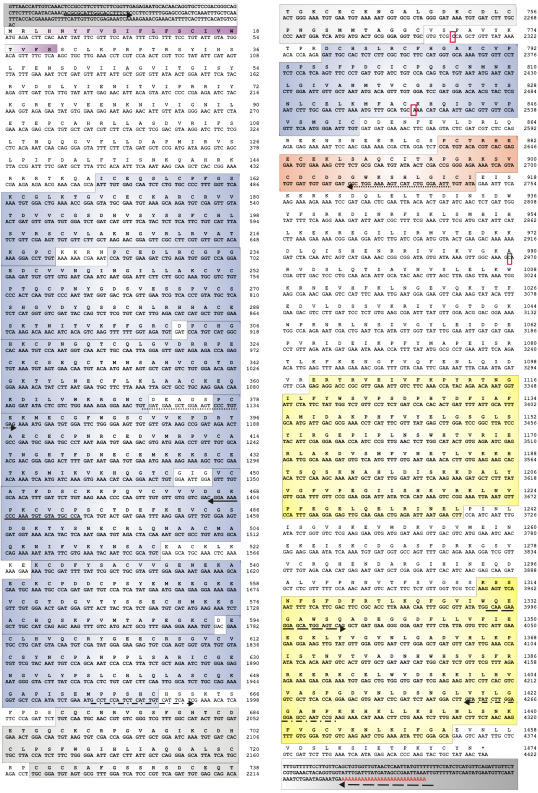
C. elegans agrin DNA and protein sequence with predicted domain architecture. The *C. elegans* agrin coding sequence was assembled from overlapping cDNA fragments, amplified by RT-PCR. The positions of the primers are shown by black arrows, where the corresponding pairs are depicted with the same line pattern (full line, dotted line, “dash-dot-dash” line). The three nucleotides missing in the genomic sequence of the database entry are framed with red rectangles. Based on the nucleotide numbering in cosmid F41G3, their positions are: C after 30028, A after 29776 and C after position 28351. The coding region of the gene is 4422 bp long with 5′ and 3′ untranslated regions of 212 and 160 bp, respectively (dark gray boxes; EMBL/GeneBank Accession AM773423). The predicted protein sequence is 1473 amino acids long and the domain architecture is shown in different colors. A putative signal sequence (purple box) is followed by seven follistatin domains (blue), two epidermal growth factor domains of the laminin-type (light gray), a follistatin domain (blue), an EGF-like domain (orange) and two laminin G domains (yellow).

**Figure 2:**
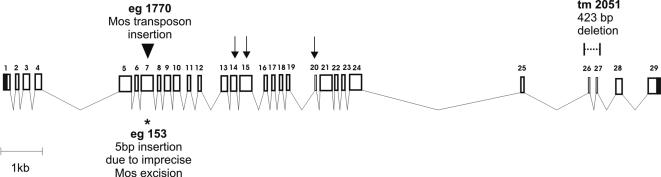
Genomic organization of the *agr-1* gene and mutant alleles. The assembled transcript consists of 29 exons which span over almost 14.5 kb on chromosome 2. Black arrows indicate the three locations where a nucleotide is missing in the database genomic sequence (exons 14, 15 and 20; cf. Fig. 1). Three mutations in the agrin gene were isolated. In the *eg1770* mutant strain (black arrowhead) *Mos1* transposon was inserted into the seventh exon which results in an out-of-frame transcript, therefore causing a putative strong loss of function mutation. The *eg153* strain (asterisk) was created by imprecise excision of the *Mos1* transposon leaving 5 bp at the insertion site and resulting in a +2 frameshift mutation. Mutant *tm2051* (dotted line) carries a deletion of 423 bp including exons 26 and 27 resulting in an in-frame loss of 42 amino acids.

A nematode agrin gene, with sequence homology to vertebrate agrin, was identified in the *C. elegans* genome. The analysis was based on queries by BLAST searches of Wormpep followed by reciprocal BLAST searches of insect or mammalian orthologs in GenBank [54]. In WormBase, the online database of the *C. elegans* genome, the agrin gene was mapped to the cosmid F41G3, originally as two separate open reading frames (ORFs) named F41G3.12 and F41G3.15, corresponding to the 5′ and the other to the 3′ part of vertebrate agrin, respectively. Based on the predicted gene sequences, the agrin-specific primers (Table 1; Fig. 1) were used to amplify overlapping fragments of each of the predicted ORFs and of a putative common transcript from cDNA reverse transcribed from RNA isolated from mixed stages of worms. As a result, three overlapping fragments gave rise to one unique agrin sequence instead of the two ORFs predicted by WormBase. The incorrect prediction was probably due to three sequence mistakes present in WormBase which resulted in false stop codons. The additional bases identified in our cDNAs are highlighted with red rectangles in Fig. 1 and their positions in the genome are indicated by black arrows in Fig. 2. In WormBase release WS170 a single agrin transcript of 1483 aa is predicted that shares 88% identity with our experimentally determined sequence. Due to the above mentioned nucleotide omissions in the genomic sequence as well as several incorrect splice site predictions the WormBase protein differs from ours in six locations. The identified *agr-1* coding region is 4422 bp long, with 5′ and 3′ untranslated regions of 212 and 160 bp, respectively (Fig. 1; EMBL/GeneBank Accession AM773423). The agrin ORF is encoded by 29 exons which span a chromosomal region of almost 14.5 kb (Fig. 2). The gene harbors two very big introns, one following exon 4 and the other following exon 24, but no alternative splicing was found. We attempted to identify putative alternative exons corresponding to the B^+^/z^+^ splicing variants described in vertebrates [20], [22] by performing PCR with primers on neighboring exons, but could not detect any.

**Table 1:** Primer sequences.

Primer name	Sequence (5′→3′ direction)
agr 1	TGATGAAGCTGGAAGTCCCTGTGAG
agr 2	AATTCCCAGATGACTTTTCCAGCC
agr 3	ACGCGCATTGGCACCTTTCTC
agr 6	TGGGCATACACATTTGGGTTTTCCG
agr 9	GCCTCCATCTCATTGTCATTCATC
agr 12	TCGGATTGGCTCCTCCAAGATATAC
agr 25	GCAAGAAGGAGCATGGAGTCAG
agr/TN (antisense)	GAAATTTCAGGCGCCATGGAGCAGTTGGGGCCTTTC
E32	AAGCATGCGGAGTTGAGTGGAGACGC
E33	AACTCTAGAGCGGCCGCAAGATCGATGTGAAGTCTCATGTCGAC
E34	CACATCGATCCTCGTCCAACTCGTTCC
E35	TAGAGCGGCCGCTTCATTATCAAAAGTCTCTCC
E36	AAATCGATATGAGTAAAGGAGAAGAAC
E37	TTATCGATCTAGGCGCCTTTGTATAGTTCATCCATGCC
E149	TTGGTACCGCATGCCCGAGAGGAGTACGCGTCC
E150	TTGAGCTCAGTCGACTGCAGCATGGCGACATGTGAAAGTGAAAATG
lam 3	GAAGCATGCATGGCGCCTGAAATTTCAAGAAC
lam 6	GTGAAGCTTAGATATCAAATTGATTGGAAGT
lam 8	GTGAAGCTTTTAGTTATAGCAGTACTTGGGT
lam 8 euk 4	CAGCGGCCGCCATCTAGATTAGTTATAGCAGTACTTGGG
oligo dT (for 3′UTR)	GGCATGGTT(TTT)_6_
overlap5′ agr (sense)	ATGGCGCCTGAAATTTCAAG
T3 primer	TTAATTGGGAGTGATTTCCC
XY1	GCCTGCAGTGTTATGAATTTTTCTTGAG
XY2	CCTCTAGATGAAAGTGAAAATGTTTCGTTTC

### AGR-1 protein domain architecture is similar but not identical to its vertebrate homologues

**Figure 3:**
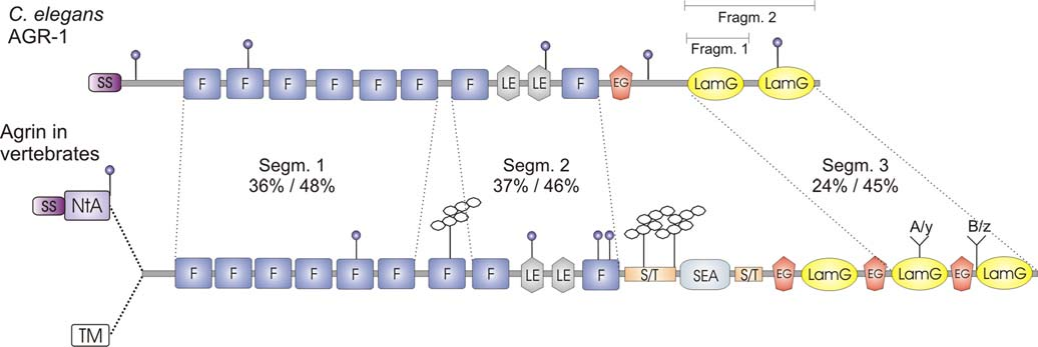
Domain architecture of the *C. elegans* agrin protein in comparison to the vertebrate orthologues. *C. elegans* agrin starts with a signal sequence (SS; purple), followed by seven follistatin-like domains (F; blue), two epidermal growth factor (EGF) domains of the laminin-type (LE; gray), one follistatin-like domain (F; blue), an EGF-like domain (EG; orange), and two laminin G domains at the C terminus (LamG; yellow). The color scheme follows the same pattern as presented in the Fig. 1. Predicted N-glycosylation sites are shown with blue circles. Vertebrate agrins have two alternative N-termini: a secreted form, with a signal sequence (SS; dark purple) and a laminin-binding N-terminal agrin domain (NtA; light purple). These are followed by follistatin domains, including one more than in *C. elegans* (F; blue), a sea urchin sperm protein, enterokinase and agrin domain (SEA; light blue), two serine/threonine rich regions (S/T; light orange), and three laminin G domains (LamG; yellow). O-linked heparan sulphate and chondroitin sulphate chains are schematically shown as branches and several N-linked glycoslation sites as blue circles. Alternative splicing at the last two LamG domains of vertebrate agrin (A/y and B/z) gives rise to several agrin isoforms with different functions, but no alternative splicing was found in *C. elegans* agrin. Three separate segments of *C. elegans* agrin marked by dashed lines were used in a Blast search. The resulting degrees of identity/similarity to the corresponding parts of chicken agrin (Swissprot entry P31696-2) are indicated. Recombinant fragments 1 and 2 of *C. elegans* agrin indicated above the LamG domains were used as antigens for raising monoclonal and polyclonal antibodies, respectively.

The predicted protein sequence of agrin (AGR-1) consists of 1474 amino acids (Fig. 1). Domains were predicted by computational analysis of this protein sequence using the SMART bioinformatics tool package (Fig. 3) [55]. A putative signal sequence of 22 amino acids (purple) is followed by seven Follistatin domains (F, blue), two epidermal growth factor domains of the laminin-type (LE, gray), another follistatin domain (F, blue), an epidermal growth factor (EGF)-like domain (EG, orange) and two laminin G domains (LamG, yellow).

When compared to the known vertebrate agrin orthologues, the *C. elegans* protein shares a high similarity in terms of modular architecture, but is missing certain domains (Fig. 3). Vertebrate agrin molecules exist in two different forms, one with a signal sequence followed by a laminin-binding NtA domain (Fig. 3, light purple) and another one with a non-cleaved signal sequence (TM, empty rectangle) serving as a transmembrane anchor [11], [18]. Using a 5′ RACE approach in *C. elegans* only one isoform was found, containing a signal sequence but no laminin-binding NtA domain. The signal sequence, together with a corresponding cleavage site between the amino acids 22 and 23, was predicted with 0.74 probability by Signal P3.0 Server [56]. However, we could not find any potential exon encoding a domain similar to the NtA domain in the genomic sequence. The N-terminal part of AGR-1 has seven repetitive follistatin domains, while vertebrate agrin contains eight of them. Further differences are present in the C-terminal part of the AGR-1 protein where the serine/theonine-rich regions (S/T, light yellow) as well as the SEA (sea urchin sperm protein, enterokinase, agrin) domain are missing. Vertebrate agrin is a heavily glycosylated protein carrying large O-linked heparan sulphate and chondroitin sulphate chains at several positions in the protein [14], [15] shown as branched structures in Fig. 3. This is probably not the case for AGR-1 since the S/T-rich region is missing. Finally, vertebrate agrins have three laminin G domains (lam G), while AGR-1 has only two and no EGF-like domains separating them. The overall similarities of the different agrin segments to the corresponding regions of chicken agrin are indicated in Fig. 3.

**Figure 4:**
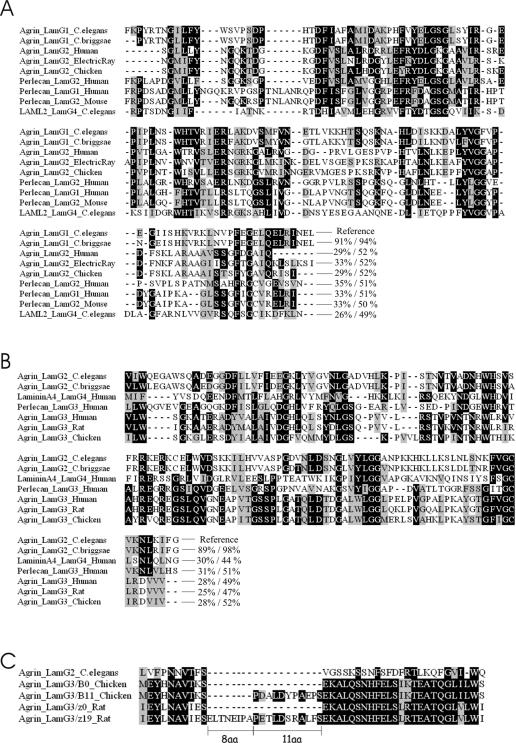
Alignment of the *C. elegans* lamG domains to the corresponding domains of other proteins. A, The first LamG domain of the *C. elegans* protein (Agrin_LamG1_*C.elegans*) was used as a query for Swissprot, Trembl and Refseq databases. After the analysis of an extensive alignment, the best hits were selected for this representation and include: the predicted agrin orthologue of *C. briggsae* (Agrin_LamG1_*C.briggsae*), the agrin LamG2 domains of the human, electric ray and chicken proteins, the LamG2 and LamG1 of human perlecan and the LamG4 of a laminin-like protein 2 (LAML2) identified in *C. elegans*. The similarities between each of the sequences compared to the *C. elegans* lamG1 are expressed as % identity/% similarity. B, The second LamG domain of the *C. elegans* protein (Agrin_LamG2_*C.elegans*) was used as a query for Swissprot, Trembl and Refseq databases. After the analysis of a more extensive alignment, the best hits were selected for this representation and include: the predicted agrin orthologue of *C. briggsae* (Agrin_LamG2_*C. briggsae*), the LamG4 of human lamininA4, the LamG3 of human perlecan and the agrin LamG3 domains of the human, electric ray and chicken proteins. The similarities between each of the sequences compared to the *C. elegans* lamG1 are expressed as % identity/% similarity. C, The *C. elegans* agrin sequence aligns best with the B_0_/z_0_ isoforms of chick and rat agrin, respectively. The conserved alternatively spliced agrin exons, encoding 8 aa, 11 aa or 19 aa inserts at this site, do not exist in *C. elegans*.

When the LamG domains from the *C. elegans* protein were used as a query against Swissprot, Trembl and Refseq databases the best hits besides the *C. briggsae* predicted agrin homologue (Q61GM7_CAEBR) included vertebrate agrins as well as laminins themselves and perlecans, shown in the alignments of Fig. 4A and 4B. In addition, a reciprocal analysis was done to see if some vertebrate agrin lamG domains produce best similarity hits with the nematode one. It turned out that the LamG domains of *C. elegans* agrin are quite distinct from those in vertebrates, and that the most similar lamG domains in vertebrates do not produce clear reciprocal best hits. This is not surprising since the LamG domains of the different proteins revealed equal similiarites to *C. elegans* agrin LamG domains (Fig. 4A,B). However, blast searches with each AGR-1 LamG separately resulted in a better match between the first *C. elegans* lamG and the second LamG from several vertebrate homologues, as well as between the second *C. elegans* LamG and the third LamG domain of vertebrate agrins (Fig. 4A and B). This implies that the two LamG domains of *C. elegans* agrin rather correspond to the last two LamG domains of vertebrate agrin. This is further supported by the alignment of the region preceding the second LamG domain of AGR-1, which aligned best to the chicken B_0_ agrin isoform, i.e. it does not contain any inserts at the conserved B/z site (Fig. 4C). Based on the overall domain architecture and the fact that *agr-1* is the only gene in the *C. elegans* genome to encode a protein of this unique domain composition we conclude that AGR-1 is the nematode agrin orthologue and not an orthologue of any other lamG domain-containing protein.

### *Agr-1* is expressed in *C. elegans* buccal epithelium, dorsal and ventral IL1 head neurons, and distal tip cells of developing gonad, but not in body wall muscles

**Figure 5:**
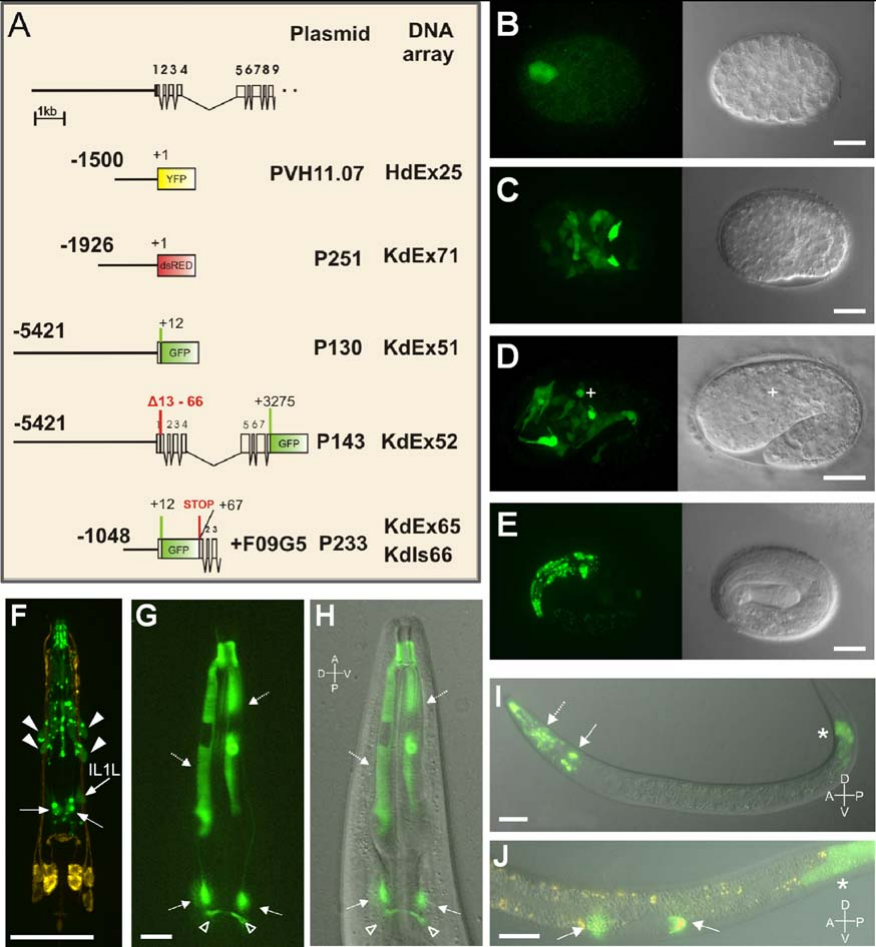
* Agr-1::reporter* expression in transgenic animals. A, Reporter genes were fused to different portions of agrin non-coding and coding sequences as shown in the schematic representation of the genomic region containing the *agr-1* promoter and *agr-1* 5′-end. The lengths of the promoter or gene sequences and the names of the the *pagr-1*::*reporter* plasmids and DNA arrays are indicated. Since all of these constructs resulted in the same expression patterns, representative micrographs of the *kdIs66* transgenic worms are shown in B–J. B Expression starts in 2 cells in the anterior part of the embryo at around the 64 AB cell stage. C, Towards the end of gastrulation expression is seen in about 40 cells throughout the embryo including neuronal precursors, several ventral hypodermal cells and pharyngeal precursor cells (ventral view). D At the 1 1/2 to 2 fold stage expression is seen in IL1 neurons (identity determined postembryonically), embryonic motoneurons and a number of additional cells in the head, most likely arcade cells and epithelial buccal cells in the pharynx, and in few apoptotic cells (marked by +). E, In the 3fold stage embryos expression is seen in the IL1 neurons (6 neurons), most of the arcade cells (3 anterior arcade cells and the DL and DR posterior arcade cells) and the buccal epithelial cells in the pharynx. The 2 lateral IL1 neurons express GFP only weakly and only in early larval stages, wheras the remaining 4 IL1 neurons express GFP strongly throughout all larval stages. F (dorsal view) and I In L1 larvae expression is observed, in the buccal epithelial cells (dashed arrow), in 3 anterior arcade cells and the DL and DR posterior arcade cells (arrowheads), and in IL1v and IL1d neurons (arrows) and posterior gut cells (asterisk). In F, the worm was co-stained with DiI. G and H Head of a young adult worm; expression is visible in the buccal epithelial cells (dashed arrows) and in the IL1v and IL1d neurons (arrows); open arrowheads point at the IL1 processes in the nerve ring. J, L2 larva; expression in the migrating distal tip cells (arrows) and posterior gut (asterisk). Bars are 10μm.

To determine the expression pattern of the *agr-1* gene, we made different *agr-1*::*reporter* fusion constructs containing up to 5421 bp upstream of the first exon and up to 3275 bp of sequence downstream of the translational start codon including the large intron after exon 4 (Fig. 5A). Another construct containing the *gfp* gene flanked by *agrin* genomic sequences was co-injected with the cosmid F09G5. For all five constructs 5–10 lines were isolated and all of them exhibited GFP expression in the same patterns. Thus inclusion of 1048 bp upstream of the transcription start seem to harbor all regulatory sequences required to direct the highly distinctive expression during development and in the adult worm. Fluorescence started to be visible in two cells of young embryos at around the 64 AB cell stage (Fig. 5B). Towards the end of gastrulation expression was visible in about 40 cells throughout the embryo including neuronal precursors, ventral hypodermal cells, and pharyngeal precursor cells (Fig. 5C). At the 1 ½ to 2 fold stages fluorescence was observed in IL1 neurons (the identity was determined post-embryonically, see below), the nine buccal epidermal cells, and additional cells in the head, most likely arcade cells (Fig. 5D). Transient expression was also observed in embryonic motoneurons (no longer visible in 3 fold stage embryos) and in a few apoptotic cells in the head. Based on their position they could be the sister cells of some of the IL1 neurons, which are known to undergo programmed cell death at this developmental stage (Fig. 5D). At the 3 fold stage expression was restricted to the buccal epidermal cells, most of the arcade cells (3 anterior and the DL and DR posterior arcade cells), and the six IL1 neurons (Fig. 5E). The two lateral IL1 neurons expressed the marker only weakly also in the L1 larval stage (but not later during development) (Fig 5 F and I), whereas the dorsal and ventral IL1 neurons expressed GFP strongly throughout all larval stages and in the adults (Fig. 5 F–I). Starting from the L1 larval stage expression could also be observed in the posterior cells of the gut (Fig 5 I–J). Starting from the L2 stage, when gonad development and migration begins, fluorescence became also visible in the distal tip cells of the gonad (Fig. 5J).

**Figure 6:**
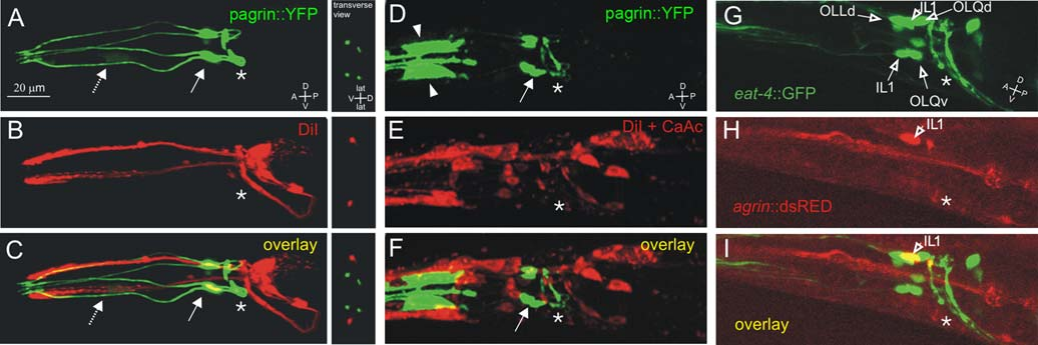
* Agr-1* expression in IL1d and IL1v neurons. A–C, DiI staining in *hdEx25* trangenic worms; no co-staining is observed between *agr-1::YFP* (A) and DiI (B). In D–F, no costaining is observed between *agr-1::GFP* (D) and DiI+CaAcetate (E) in *kdIs66* transgenic animals. In G–I, costaining is observed in *eat-4::GFP* (G) and *agr-1::dsRED* (H) in *adIs1240; kdEx71* transgenic worms. Figures C, F, I show merged channels. In all panels dashed arrows point out dendrites; arrows point to neuronal cell bodies; arrowheads mark buccal epithelial cells and asterisks indicate the nerve ring.

To identify the four head-neurons expressing *agr-1*, we stained amphid neurons with DiI in the *hdEx25* transgenic lines, with or without 50mM CaAcetate. We did not find any co-staining between the red and yellow fluorescing dyes in these experiments, suggesting that the four neurons were neither amphids, nor IL2 neurons (Fig. 6A–F). We then analyzed *agr-1::dsRED* expression in the *kdEx71; otIs107(ser-2::gfp)* [57] and in the *kdEx71; adIs1240(eat-4::gfp)* [58] transgenic lines. No co-staining was observed in *kdEx71; otIs107* (results not shown), suggesting that the neurons expressing *agr-1* are not the OLL neurons. In the *kdEx71; adIs1240* animals the dorsal and the ventral, but not the lateral IL1 neurons co-stained for the red and green markers, allowing us to identify the *agr-1* expressing neurons as the dorsal and ventral IL1 neurons (Fig. 6G–I).

In vertebrates agrin plays an important role in synaptogenesis at the neuromuscular junctions and in muscle stability (reviewed in [8]), therefore we carefully looked whether *agr-1* is expressed in muscles and neurons of the nematode. We did not observe any marker expression in muscles, and we observed a weak, transient expression in motoneurons in the embryo, but not in larval nor adult stages, implying that the gene is not expressed in these tissues postembryonically or is expressed at undetectable levels.

### *Agr-1* does not interact genetically with genes important for synaptogenesis and muscle stability in the worm

To further investigate the putative involvement of *agr-1* in synaptogenesis and muscle stability, we tested potential genetic interactions between *agr-1* and factors involved in these processes in the worm (summarized in Table 2). The *agr-1(eg1770)* single mutant did not show any obvious phenotype and its movements and co-ordination seemed normal. We tested the sensitivity to aldicarb and levamisole of the *agr-1(eg1770)* single mutant and in the *lev-1(e211)* [59] and *dys-1(cx18); dyb-1(cx36)* [60], [61] backgrounds. The *eg1770* mutation did not influence cholinergic activity in these backgrounds suggesting that the *agr-1* gene does not play a role in the biogenesis and activity of cholinergic synapses (data not shown).

**Table 2:** Different genetically sensitized backgrounds did not reveal a function for *agr-1*.

*agr-1* locus comparison	genetic background	phenotypes tested
eg1770 vs. WT	WT	a, b, c, d, e, f, n, h, s
	*lev-1(e211)*	a, b, c, d
	*dys-1(cx18)*	a, b, c, d, g
	*dys-1(cx18)*	a, b, c, d, g
	*dys-1(cx18); dyb-1(cx36)*	a, b, c, d
	*hlh-1(cc561)*	a*, b*, c*, d*, f*
	*unc-52(e444)*	a, h, i, r, d
	*unc-52(gk3)*	a, h,i, d
	*cle-1(cg120)*	a, b
	*nid-1(cg119)*	a, b
	*dig-1(n1321)*	a, b, p, q
*eg153* vs.WT	WT	a, b, c, n, s
*tm2051* vs. WT	WT	a, b, s

*Agr-1* mutants were tested in different genetic backgrounds and the following phenotypes were analyzed: a, locomotion; b, response to touch on head and tail; c, sensitivity to levamisole; d, sensitivity to aldicarb; e, muscle integrity by DIC; f, muscle integrity by rhodamine-phalloidin staining; g, muscle integrity (myosin fibers) in worms expressing MYO-3::GFP (stEx30); h growth rate; i, paralysis progression; m, brood size, n, thrashing assay; p, egg laying; q, gonad displacement; r, gonad arm migration; s, pharynx pumping-rate in feeding worms. *, tested at 15°C and at 20°C.

In order to investigate possible genetic interactions of *agr-1* with components of the dystrophin-glycoprotein-complex (DGC) we generated double and triple mutants between *agr-1(eg1770)* and the following mutant strains: dystrophin *dys-1(cx18)*, dystrobrevin *dyb-1(cx36)*, *dys-1(cx18);dyb-1(cx36)* [60], [61], as well as with *hlh-1(cc561)*, previously shown to enhance the phenotypes of hypomorphic muscle mutants due to a mutation in the transcription factor MyoD1 [62]. In addition, *agr-1(eg1770)* was crossed with several mutants for components of the extracellular matrix, i.e. perlecan *unc-52(gk3)*, *unc-52(e444)* [63], collagen *cle-1(cg120)* [64] and *nidogen nid-1(cg119)* [65], and with a mutant affecting the cell adhesion factor *dig-1(n1321)* [66]. The single, double, and triple mutant strains were scored for locomotion in normal conditions or under stress, in thrashing assays, response to touch, and viability. In addition muscle integrity was assessed by visualizing the muscle fibers either with rhodamine-phalloidin staining [67] or with the *stEx30(myo-3::gfp)* array [68]. The *agr-1(eg1770)* mutation did not aggravate any of the phenotypes of the different mutant backgrounds, suggesting that *agr-1* function is dispensable in the muscles of the worm (Table 2).

### Specific monoclonal and polyclonal antibodies detect agrin in the basement membrane of the pharynx

**Figure 7:**
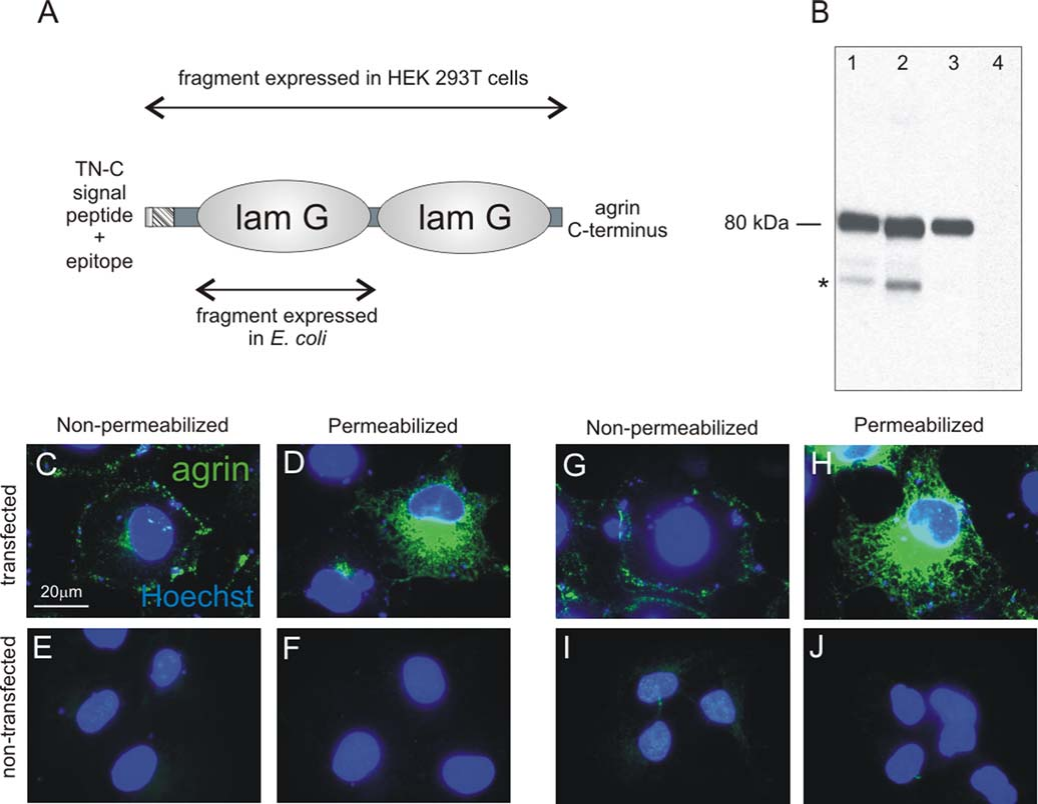
Antibodies against *C. elegans* agrin. A, Schematic representation of the recombinant fragments used as antigen to raise monoclonal and polyclonal antibodies. For eukaryotic expression the C-terminal lamG domains were fused to a short fragment of chicken tenascin C (Tn-C), including a secretion signal and the epitope of the anti-Tn60 antibody. The specificity of both polyclonal and monoclonal antibodies was tested by western blotting on conditioned medium of COS cells transfected with the construct encoding the two LamG domains with the Tn-C-tag (B). Lanes 1 and 2 were incubated with polyclonal antisera from two different rabbits, lane 3 with the monoclonal antibody pool raised against the bacterially expressed fragment, and lane 4 with pre-immune serum. All antibodies detected a band of about 80 kDa, which corresponds to the size of the recombinant protein. Additional smaller bands (asterisk) most likely correspond to degradation products which are not recognized by the monoclonal antibodies. C–J, Immunofluorescence staining of transfected COS cells was performed with the anti-agrin monoclonal antibody pool (C–F) and compared to the anti-Tn60 control (G–J). In transfected cells (C and D; G and H) the secreted agrin fragment was detected on cell surfaces of non-permeabilized cells (C and G) or in the endoplasmatic reticulum/Golgi apparatus of permeabilized cells (D and H). Non-transfected cells were used as a negative control (E and F; I and J).

To confirm the agrin expression pattern at the protein level and to obtain more information on its possible function in the nematode, we raised specific antibodies against the lamG domains (Fig. 7A). The first lamG domain was expressed in *E. coli*, purified and used as antigen to raise monoclonal antibodies. A fragment containing both lamG domains was fused to a short sequence of chicken tenascin-C (Tn-C), expressed in HEK293EBNA cells, purified from the conditioned media and used to raise polyclonal antibodies. The specificity of both polyclonal and monoclonal antibodies was tested by western blotting and immunostaining of COS cells transiently transfected with the TN-C-agrin fusion construct (Fig. 7). In the conditioned medium, a band of around 80 kDa, which corresponds to the size of the recombinant protein, was detected by all antibodies. The polyclonal serum recognized additional smaller fragments which probably correspond to protein degradation products. Transfected and non-transfected cells were stained with the anti-agrin monoclonal antibody pool (Fig. 7C–F) as well as the monoclonal antibody against the TN-C epitope tag (Fig. 7G–J). In the permeabilized cells (Fig. 7D and H), the overexpressed fragment could be detected mainly in the Golgi apparatus while in non-permeabilized cells (Fig. 7C and G) the agrin protein could be detected in patches bound to the plasma membrane. This represented a first indication that COS cells may express a surface receptor which binds agrin. Non-transfected COS cells did not give any immunfluorescence signal (Fig. 7 E–J).

**Figure 8:**
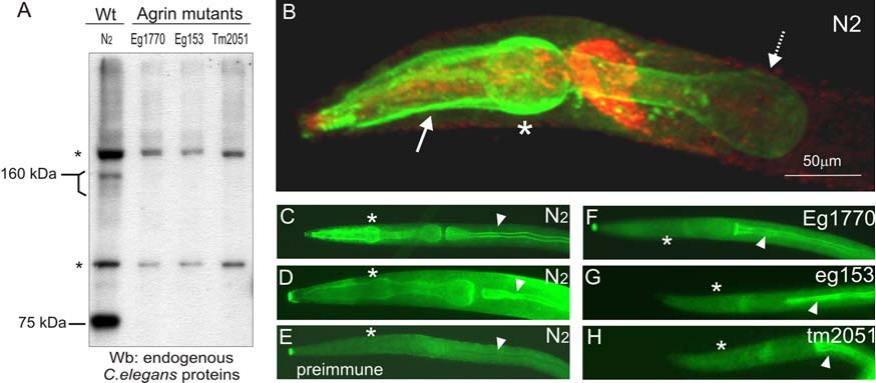
Detection of endogenous *C. elegans* agrin by western blot and immunofluorescence. Lysates of wild type (N_2_) and agrin mutant worms (*eg1770*, *eg153*, *tm2051*) were analysed on western blots (A). Two prominent bands of about 160 kDa and 75 kDa were present exclusively in the wild type (Wt) worms and not the mutants. The larger band corresponds to the calculated size of the full length AGR-1 protein and the smaller band may represent an agrin degradation product. Asterisks denote two additional background bands present in all the strains. B, Worms were immunostained with the monoclonal antibody pool against *C. elegans* agrin (green) and Rim, a synaptic marker prominent in nerve ring (red). Agrin was detected in the basal lamina around the pharynx procorpus (arrow) and anterior bulb (asterisk). Posterior bulb staining was weaker possibly due to poor antibody penetration (dashed arrow). (C–H) Polyclonal antiserum staining resulted in the same pattern in the pharynx of wild type worms (C and D, asterisk for anterior bulb) whereas it was clearly absent in agrin mutants (F–H). Prominent background staining of the gut was present in all strains (C–H, arrowhead). Preimmune serum of the same rabbit was used as negative control on wild type worms (E) where both pharyngeal and gut staining was clearly missing.

In order to detect the endogenous worm agrin protein, we analyzed worm extracts by western blotting with purified anti-agrin polyclonal antibodies. We compared extracts from wild type worms with the three *agrin* mutants available, *agr-1(eg1770), agr-1(eg153)* and *agr-1(tm2051)* described in Experimental procedures. Extracts were prepared from about 50 µl of synchronized young larval stages (L1 and L2). The fractions soluble in RIPA buffer (including 6M urea) are presented in Fig. 8A. Two bands, one of about 160 kDa and the other of about 75 kDa, were detected by anti-agrin antibody in the lysate of wild type worms, but not in any of the three *agrin* mutant strains. The 160 kDa band corresponds to the calculated weight of the full length agrin protein, while the smaller band could represent a degradation product. Two additional bands (asterisks) which were present in all analyzed strains seem to be due to a cross-reactivity with other unidentified proteins.

To localize the agrin protein, the worms were co-stained with the monoclonal antibody pool against *C. elegans* agrin and with polyclonal anti-rim antibody, recognizing a pre-synaptic marker prominent in the nerve ring [69]. The major site of agrin expression was around the pharynx and the staining was particularly enriched in the anterior part (Fig. 8B). The posterior bulb was labeled more weakly correlating with the fainter GFP reporter expression in the posterior part. Polyclonal antiserum staining resulted in the same staining pattern in wild type worms of different developmental stages (Fig. 8C and D). Young larvae (L1) generally showed stronger agrin staining compared to young adults (C compared to D, respectively). Pharyngeal staining was absent in all three agrin mutant strains, which is an additional confirmation for their lack of agrin expression (Fig. 8F–H). No staining could be observed in body wall muscles, in the synapses along the ventral and dorsal nerve cords, in the gonad and in the posterior gut cells. In addition to the pharynx staining in the wild type worms, the polyclonal antiserum stained the gut lumen (arrowhead) both in the wild type worms as well as in the agrin mutants, but not when preimmune serum was used. The staining of the lumen of the gut represents an unrelated cross-reactivity of our antiserum, possibly corresponding to the background bands detected on the western blots.

The strong pharyngeal localization led us to suspect that agrin has a pharynx-related function, namely in feeding behavior or structural stability. Therefore, we investigated the pharyngeal pumping rate of normal versus mutant animals, but could not detect any differences. Despite the absence of agrin in the pharynx of the mutants, pharyngeal morphology was normal in young as well as in older animals (data not shown). To challenge the pharynx function, we fed agrin mutants with different strains of bacteria of various sizes [70]. However, even ingestion of the largest bacteria, strain *Bacillus megaterium*, did not result in a different growth rate as compared to the wild type animals.

### Recombinant fragment of *C. elegans* agrin binds to purified chicken α-dystroglycan

**Figure 9:**
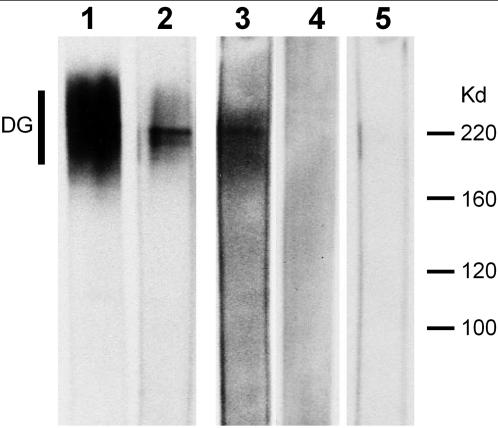
* In vitro* interaction between *C. elegans* agrin and vertebrate α-dystroglycan. Purified chicken α-DG (lanes 1–3 and 5) or crude COS cell extract (lane 4) was transferred to the membrane after separation by SDS-PAGE and membrane strips were incubated with different samples of agrin: lane 1, chicken muscle agrin isoform; lane 2, chicken neuronal isoform; lanes 3 and 4, *C. elegans* agrin in conditioned medium of transfected COS cells; lane 5, conditioned medium of non-transfected COS cells. Binding of the respective agrins was detected by anti-chick agrin antibody (lanes 1 and 2) or with the Tn60 antibody recognizing the short tenascin C fragment which was fused to the *C. elegans* agrin fragment (lanes 3, 4 and 5). Binding of *C. elegans* agrin to α-DG was detected in lane 3, but not in the negative controls (lanes 4 and 5).

Based on the well described binding of agrin to α-dystrolgycan in vertebrates [31], [32], we addressed this possible interaction in the case of *C. elegans* agrin. The interaction between agrin recombinant fragment containing two lamG domains and dystroglycan was tested biochemically by a protein overlay assay. Purified chicken α-dystroglycan was run on an SDS-acrylamide gel and blotted to a nitrocellulose membrane. Strips of this membrane were incubated either with conditioned medium containing the recombinant *C. elegans* agrin fragment or with purified recombinant chicken agrin fragments representing the muscle (A_0_B_0_) and neuronal (A_4_B_8_) agrin isoforms. Following several wash steps, agrin bound to α-DG was detected with anti-Tn60 antibodies that recognize the short TnC part fused to agrin (Fig. 9). The chicken muscle agrin fragment, which served as positive control bound efficiently to dystroglycan, appearing as a dark smear caused by the migration behaviour of the highly glycosylated distroglycan (Fig. 9, lane 1), while the neuronal isoform gave a very weak signal at the dystroglycan protein core size of approximately 200 kD (lane 2). The *C. elegans* agrin fragment also bound to DG and was detectable as a smear on the membrane strip similar to the chicken muscle agrin (Fig. 9, lane 3). In parallel, a strip containing 50 µg of proteins of crude COS cell lysate served as negative control for unspecific binding of agrin to any blotted proteins (Fig. 9, lane 4). Furthermore, dystroglycan containing strips incubated with conditioned medium from non-transfected cells did not result in a signal (lane 5). This indicates that *C. elegans* agrin harbors the ability to specifically bind to α-dystroglycan.

### *C. elegans* agrin induces endogenous dystroglycan clustering in COS cells

**Figure 10:**
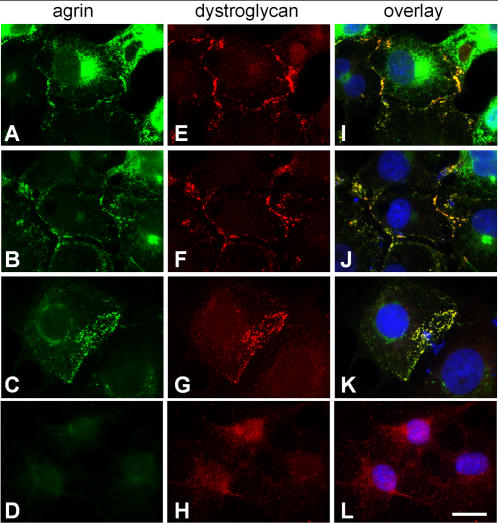
Recombinant *C. elegans* agrin clusters endogenous dystroglycan in COS cells. COS cells were transfected with the recombinant fragment of *C. elegans* agrin and immunostained for agrin and endogenous β-DG. A–D, agrin staining; E–F, anti-β-DG staining; I–L, overlay including nuclear staining. A–C, In transfected cells, secreted agrin bound to the cell surface in a patchy pattern to cells producing large quantities of agrin (A) as well as to cells expressing little or no agrin themselves (B, C). The cells were co-immunostained for endogenous β-DG (E–H) which, in transfected cell cultures, colocalized with agrin on cell surfaces. In non-transfected cells (D–L), no agrin staining was present (D) and β-DG showed diffuse staining (H and L).

The interaction between *C. elegans* agrin and DG was further investigated in cell cultures of COS cells transfected with the recombinant fragment of *C. elegans* agrin (fragment 2 in Fig. 3). Transfected cells secreted the agrin fragment into the medium where it bound to the cell surfaces of transfected as well as untransfected cells in a patchy pattern (Fig. 10 A–C). Remarkably, the dystroglycan staining followed the same pattern (Fig. 10 E–G) and overlapped with the agrin staining in patches (Fig. I–K), while the dystroglycan staining of untransfected cells showed a diffuse and intracellular staining (Fig. 10 H). The anti-β-DG antibody recognizes the intracellular part of the protein, therefore only permeabilized cells show strong staining. Immunostaining experiments with the pool of the monoclonal anti-agrin antibodies resulted in the same pattern as with polyclonal antibodies (data not shown), but co-staining with anti-β-DG was not possible due to the same host species in which the antibodies were raised. Our results suggest that the recombinant agrin fragment containing the two lamG domains bound to the cell membrane, probably through direct interaction with dystroglycan. Interestingly, the endogenous dystroglycan had a diffuse pattern in cells without agrin overexpression, but it appeared clustered by agrin secreted from the transfected cells, suggesting that agrin bound to the cells and induced clustering of DG.

## Discussion

We have identified and characterized the first invertebrate agrin. In terms of domain structure, a high degree of similarity was found between *C. elegans* and vertebrate agrins, although some domains were missing. In the worm, only one N-terminal variant was detectable containing a secretion signal but no NtA domain [11]. Several other domains were missing, namely the Ser/Thr-rich region, the SEA module, and one of the three lamG domains at the C-terminal end of the molecule. We analyzed the two lamG domains present in the worm and compared them to the vertebrate lamG domains to determine whether they corresponded to a particular domain pair of vertebrate agrin. Blast searches revealed that the AGR-1 LamG domains aligned best to the last two LamG domains of vertebrate agrin. However, we could not detect any small inserts known to be important for the clustering function of the vertebrate neuronal agrins [20], [21]. When we searched the intron sequences between the last few exons encoding the LamG domains in all three frames, we could not detect any potential alternative exons coding for amino acids resembling the conserved inserts in the A/y and B/z sites of vertebrates. Therefore, we concluded that in *C. elegans* there is only one major agrin isoform expressed and that the A/y and B/z alternative spice sites are specific to vertebrates.

With the goal to detect the endogenous agrin by western blot, we raised polyclonal antibodies against the fragment comprising two lamG domains. Purified antibodies detected protein bands of about 160 kDa and 75 kDa, present only in the lysates of the wild type worms and not in any of the agrin mutants. The bigger band corresponds to the protein core size and the smaller possibly to a degradation product or a shorter isoform. In the loss of function mutants *agr-1(eg1770)* and *agr-1(eg153)* we expected a complete lack of the protein, but *agr-1(tm2051)* carrying an in-frame deletion and was expected to express a shorter protein. However, in the *agr-1(tm2051)* mutant the deleted exons 26 and 27 encode the majority of the region against which the polyclonal antibodies were raised. Therefore, this truncated protein may not be recognized and no band detected even though the protein may be expressed. Another possibility is that the protein with the deletion does not fold properly and gets degraded. In any case, it is clear that the agrin mutants do not express the normal agrin protein as the wild type worms do.

The most surprising result of our study was the fact that we could not detect any agrin expression neither in body wall muscles nor in motoneurons innervating them (except for a transient expression in some embryonic motoneurons). The lack of agrin expression at the NMJs suggested that agrin may not have a conserved function in AChR clustering in the worm. Nevertheless, we investigated the possible phenotypes in *C. elegans agr-1* mutants related to muscle and/or NMJ function. In the case of defective muscle or NMJ formation, worms should display uncoordinated (*unc*) movement, defective thrashing pattern, and/or egg laying deficiency. However, we did not observe any significant impairment neither in *agr-1* single mutants, nor in combination with other related mutations. Pharmacological assays with levamisole, a potent nematode-specific cholinergic agonist [47] and aldicarb, an ACh esterase inhibitor [71], did not reveal any resistance in *agr-1* mutants. Therefore, in *C. elegans* we could not find any evidence for a role of agrin in muscle or synaptic functions, suggesting that the ancestral function of Agrin was not in NMJ formation. In accordance with our hypothesis, no clear MuSK orthologue (the vertebrate receptor for agrin) has been found in the nematode. Up to date only two factors important for nicotinic AChR clustering have been characterized in the worm. The Ror RTK Cam-1 is necessary for the localization of the ACR-16 (nicotinic AChR α-subunit) at the NMJ, but no kinase activity is needed for this function [51]. Another protein, essential for clustering levamisole-sensitive AChR at the NMJ in the worm, is LEV-10. Also for LEV-10, its extracellular domain was shown to be sufficient for the clustering [48]. Interestingly, vertebrates express proteins that share high similarity to CAM-1 and LEV-10, suggesting that these novel factors discovered in the worm could have been conserved during evolution. Thus, both CAM-1 and LEV-10 may be components of distinct pathways important for AChR clustering in nematodes that may have been complemented by the agrin-MuSK pathway during evolution in vertebrates.

We then took a closer look at the sites of agrin expression visualized by reporter genes and antibody staining. Prominent expression was found in four head neurons and some pharyngeal cells. This relatively restricted expression pattern was confirmed by several reporter constructs with varying portions of the gene as well as by antibody staining.

We identified the four agrin-expressing neurons by injecting *agr-1::dsRED* construct into the transgenic lines expressing GFP in specific neuron subtypes [58]. This approach identified the agrin-expressing neurons as inner labial (IL1) sensilla polymodal neurons. These are mechanosensory neurons, motoneurons and interneurons at the same time [72]. There are six IL1 neurons in total (three pairs) in the head of the worm [73], but agrin was found to be expressed only in the dorsal and ventral pairs. Such a sub-specialization might be significant to distinguish very fine sensory inputs from the environment. It is known that IL1 neurons together with OLQ (outer labial quadrant sensilla) neurons are responsible for the sensing of a light nose touch and the regulation of spontaneous foraging movements [72]. Therefore, we tested agrin mutants for light nose-touch-avoidance by the eyelash test [74]. Agrin mutants seemed to be as sensitive to touch as the wild type worms, suggesting that absence of agrin does not lead to a complete failure of IL1 function. Furthermore, IL1 neurons did not show any morphological abnormalities in the *agr-1(eg1770); hdEx25* strain.

The pharyngeal cells expressing agrin belong to the buccal epithelium which surrounds the anterior-most part of the pharyngeal lumen ([75]; Wormatlas, http://www.wormatlas.org/). In early larvae agrin is also expressed in other pharyngeal cells, most likely the marginal cells. The epithelial tissue forms a rigid narrow cylinder restricting the food entry into the pharynx [75]. Agrin expressed in this tissue might have a function in the structural support of the pharynx. The immunostaining with monoclonal and polyclonal anti-agrin antibodies detected the protein around the pharynx, resembling perlecan immunostaining in the pharynx basal lamina [76]. Thus, agrin is probably secreted from the pharyngeal cells and integrated into the basal lamina. This pharyngeal staining was missing in all three agrin mutant strains. To test proper pharynx function we measured the pumping rate, which did not differ between agrin mutants and wild type worms. To challenge pharynx function more drastically we tested whether bacterial size had any influence on the feeding and ingestion capability of the worms. For that purpose we grew the worms on *Bacillus megatherium*, a strain of large bacteria and compared it to the *E. coli* OP50 strain used in regular laboratory maintenance [70]. Pumping defective mutant strains *eat-4(ky5)* and *eat-5(ad464)*, which grow much more slowly on large bacteria, were used as positive controls. On both food sources our agrin mutant *agr-1 (eg1770)* grew equally fast as wild type worms. Pharynx morphology and resistance to mechanical stress seemed normal in the *agr-1 (eg1770)* mutant animals, suggesting that agrin is not essential for proper development or structural support. If agrin plays a role in pharynx-related functions, it is subtle or redundant.

Some expression of agrin was present in the distal tip cells of developing gonads in young larvae. The distal tip cell is critical for germ line proliferation and guides gonad migration by sensing environmental cues [77], [78]. We have investigated a possible effect of agrin deficiency in gonad migration or brood size, but the mutants were indistinguishable from the wild type worms in these aspects.

Interaction between agrin and α-dystroglycan in vertebrates is important in different tissues and processes (reviewed in [8], [79]). At the NMJ, DG stabilizes mature synapses by connecting the basal lamina to cortical F-actin. In the muscle sarcolemma DG is a central component of a large dystrophin-glycoprotein complex (DGC) where it serves again as a linker between the ECM and intracellular cytoskeletal proteins (reviewed in [33], [34]). At the NMJ and at the muscle membrane agrin binds with its C-terminal lamG domains to α-DG and with its N-terminal part to laminin providing additional support. Interaction between agrin and α-DG is found as well in the immune system, where this binding mediates lymphocyte activation via a lipid raft-dependent mechanism [40]. Based on the established knowledge on vertebrate agrin and DG, we decided to investigate whether this interaction is conserved in *C. elegans*. We could demonstrate direct binding of a recombinant fragment of *C. elegans* agrin to purified vertebrate α-DG by a membrane overlay assay. In COS cells transfected with the same recombinant fragment, endogenous DG was clustered and colocalized with agrin bound to the cell membrane.

Taken together, our experiments suggest that the interaction between agrin and dystroglycan may be conserved in *C. elegans* but this interaction does not play a role at the NMJ or in muscle in general. This view is supported by the observation that *C. elegans* dystroglycan, *dgn-1*, is not expressed in muscle either, but rather in epithelia and neurons. It is found in basement membrane surfaces and is not involved in muscle function [80]. Therefore, the structural organization at the molecular level of the NMJ, as well as the DGC complex, is clearly distinct in *C. elegans* as compared to vertebrates. Our studies demonstrate that the existence of protein orthologs in evolutionarily distant organisms [54] does not necessarily imply identical functions, at least not in every aspect. In *C. elegans* it appears that the muscle basal lamina depends rather on the presence of the perlecan orthologue *unc-52* [63], [76], [81], [82] and on the integrin chain orthologues *pat-2* and *pat-3* [83]–[86] and not on dystroglycan and its ligands [80]. On the other hand dystroglycan and agrin appear to function in epithelia and certain neurons but not in muscle. Our results indicate that the ancestral agrin function included dystroglycan binding but not AChR clustering activity at NMJs, a feature acquired later during evolution. Since also in vertebrates, agrin is expressed outside of muscle and NMJs, *C. elegans* can be a good model organism to delineate these ancestral functions of agrin that may still be functional in vertebrates as well.

## Materials and Methods

### C. elegans culture conditions and preparation for RNA and protein extraction

The worm strains were grown at 20°C on NGM agar plates seeded with *E. coli* OP50 [41]. For growth in large amounts and further protein extraction, worms were grown at 20°C on 10 cm culture dishes with NGM medium and the addition of egg yolk [87]. In order to prepare synchronized cultures of young larvae, gravid worms were washed with M9 buffer and subjected to sucrose flotation [88], then bleached [89] followed by extensive washing in M9. The larvae hatched and, when they reached the L2 larval stage, were rinsed off the plates with M9 buffer, washed in M9, deionized water, and quick-frozen in liquid nitrogen.

### Sequence identification and cDNA cloning

Total RNA was isolated from a mixed-stage worm population (Trizol reagent, Gibco) and reverse-transcribed into cDNA using oligo dT primers and MMLV reverse transcriptase from Advantage™ RT-for-PCR Kit (Clontech) according to the supplier's protocol. Agrin cDNA was amplified by using sets of primers designed according to the predicted gene sequences in the Wormbase (ACeDB: F41G3.15 and F41G3.12, as of January 1, 2003; presently, only one common ORF exists in the database under the name F41G3.12) in order to obtain overlapping PCR products using the following primers: agr3 and agr6; agr1 and agr2; agr9 and agr12; agr25 and oligo dT primers listed in Table 1. In parallel, a commercial *C. elegans* cDNA libray (OriGene Technologies) and the EST clone Yk1264e03 (vector pME18S-FL13, kindly provided by Dr. Yuji Kohara) were used as templates. The 5′UTR was determined with 5′RACE approach (Roche) following the supplier's protocol. Agrin cDNA sequence was assembled from overlapping fragments resulting in an open reading frame (ORF) of 4422 bp.

### Protein architecture analysis and alignments with vertebrate orthologues

Protein architecture was analysed with SMART (EMBL, [55]), and with Blast at ExPASy [90]. Several overlapping domains were predicted, but in this report only a representative structure is presented based on the similarity to the vertebrate orthologues. Laminin G domains of *C. elegans* agrin were used as a query in myHits [91] against Swissprot, Trembl and Refseq. After the analysis of a more extensive alignment, the most informative Swissprot hits were selected. Visualization was done in Jalview, using Zappo colors without conservation threshold, to analyze subgroups [92]. Alignments were submitted to the Boxshade server at Pasteur (http://bioweb.pasteur.fr/seqanal/interfaces/boxshade.html) for producing the greyscale shading. Pairwise comparison of sequences (% identity/% similarity) was performed using Smith Waterman alignments (as implemented in water, a tool in the EMBOSS package; [93]. The homology searches between fragments of *C. elegans* agrin sequence and chicken agrin were done at myHits (SIB) using iterative PSI-BLAST searches [91]. The *C. elegans* fragment composed of 2 lamG domains yielded different Blast scores for alternatively spliced chicken agrin isoforms (Swissprot), which was due to the presence, or absence, of the spliced exons. The alignments of the conserved alternative splicing site were produced by Blast at ExPASy.

### Agr-1::reporter expression constructs for expression pattern analysis

Agrin fragments were cloned following standard procedures [94]. The constructs used to create transgenic animals were the following:

*Pagr-1::dsRED* (p251). A 1926 bp genomic DNA fragment immediately upstream of the *agr-1* ATG start codon was amplified using the primers E149 and E150 (the sequences of the primers are listed in Table 1). The *SalI* site near the start codon was removed by introducing a point mutation in the sequence of primer E150 (bold and underlined in Table 1). The PCR fragment was then cloned into the *SphI-SalI* sites of the vector pVH14.05. The vector contained the *dsRED* gene and was constructed by cloning *dsRED* into pVH20.01 via *AgeI* and *MfeI* (pDsRed2-N1)/EcoRI(pVH20.01) giving rise to a promoterless vector with DsRed2 carrying Amp resistance.

*Pagr-1::yfp* (pVH11.07). A 1527 bp genomic DNA upstream of the *agr-1* ATG start codon was amplified with the primers XY1 and XY2 and cloned into the *PstI-XbaI* sites of pVH20.01 (promoterless *yfp* vector), kind gift for Prof. Harald Hutter. PVH20.01 was constructed by replacement of the *KpnI*-*SpeI* fragment in pPD95.75 by the same fragment from pPD132.102, promotorless vector with YFP (gf42 A.Fire).

*Pagr-1::gfp* (5.5 Kb) (p130). A 5431 bp genomic DNA fragment including 5421 bp of promoter sequence and the first 12bp of the *agr-1* ORF was amplified with the primers E32 and E33. The fragment was then ligated into the *SphI-XbaI* sites of the pPD95.75 vector (Fire Lab vector kit). The *NotI* and *ClaI* sites introduced with the primer E33 are needed for further cloning (see below).

*Pagr-1::gfp* (8.6 Kb) (p143). A 3198 bp genomic DNA fragment from the 5′ region of the *agr-1* gene was amplified with the primers E34 and E35 and cloned into the *ClaI-NotI* sites of the *pagr-1::gfp* (5.5Kb) plasmid. The *pagr-1::gfp* (8.6Kb) plasmid contains 5421 bp of promoter region and the *agr-1* genomic region covering the first seven exons and introns (the primer E35 primes on the 5′end of exon 8). Furthermore, primer E34 was chosen downstream of the putative signal sequence on exon1; the signal sequence is therefore not present in the construct.

*Pagr-1::gfp::agr-1* (p233). The *gfp* ORF was amplified from pPD95.75 with the primers E36 and E37 and ligated into the *ClaI* site of *pagr-1::gfp* (8.6Kb). A TAG stop codon terminates translation at the end of the *gfp* ORF. Orientation and sequence of the insert were checked. The *gfp* gene flanked by *agr-1* sequences was then excised from the plasmid with *AflII* and *SgrAI*, gel purified, and co-injected (20 ng/µl) with the cosmid F09G5 (100 ng/µl).

### Transgenic strains

The *agr-1::reporter* constructs were injected at a concentration of 50 ng/µl in wild type N2 animals or in *dpy-20(e1282).* As markers, a plasmid containing a *dpy-20^+^* genomic fragment (primers E42 and E43, plasmid p133) or pRF4 (*rol-6(su1006)*) were co-injected at a final concentration of 10 ng/µl [95]. Integration of the extrachromosomal arrays was induced by UV294 irradiation and the integrated strains were then backcrossed 10 times in N2 to remove unwanted mutations.

### Agrin mutant strains

Three mutations in the agrin gene were isolated. The *agr-1(eg1770)* and *agr-1(eg153)* mutants were created by *Mos*-driven mutagenesis and were kindly provided by Dr. Jean-Louis Bessereau [96]. In the *agr-1(eg1770)* mutant strain the *Mos1* transposon was inserted into the seventh exon (after the base pair at the position 4948, according to the numbering in the cosmid T13C2) which brings the transcript out of frame, therefore causing a strong loss of function mutation. We tested whether alternative splicing could result in the excision of the transposon and give rise to a functional *agrin* transcript in the *eg1770* mutants. In the cDNA from *eg1770* worms we only found an *agrin* transcript containing the transposon and no shorter forms, indicating that *eg1770* is indeed a loss of function mutant. The *agr-1(eg153)* agrin mutation was created by transposon mobilization which led to an imprecise excision and left an insertion of five base pairs (“TGATA” at the position after 4948 of cosmid T13C2) from the *agrin* ORF and gave rise to another putative out-of-frame mutation. The third mutant, *agr-1(tm2051)*, was kindly provided to us by the National BioResource Project, the Japanese *C. elegans* knock-out consortium. The mutant carries an in-frame deletion of 423 bp including exons 26 and 27 (nucleotides 22165-22587 of cosmid F41G3), which encode the last laminin G domain and therefore disrupt a part of the gene encoding potentially important sites of the protein [97].

### Analysis of agrin mutants

Agrin mutants were analyzed for potential defects at the neuromuscular junction, by pharmacological treatments with aldicarb and levamisole, following previously described procedures [47], [71].

As AGR-1 is expressed in pharynx, we analysed worms for related phenotypes, as described previously: pharyngeal pumping rate, pharyngeal morphology [98], and worms' feeding on large bacteria [70].

### Staining of amphid neurons

Amphid neurons were stained with 1,1′-dioctadecyl-3,3,3′,3′-tetramethylindocarbocyanine perchlorate (DiI, Invitrogen D-282) [99]. Young adult hermaphrodites were incubated in M9, 5 µg/ml DiI (with or without 50 mM CaAcetate) for two hours at room temperature, washed three times in M9 and transferred to a NGM plate with *E. coli* OP50. After two hours the worms were mounted on agarose pads with 30 mM Na Azide [71] and analyzed with a Zeiss LSM510 confocal microscope.

### Cloning, expression and purification of agrin fragments

Agrin cDNA coding for the first laminin G domain was amplified with primers containing restriction sites: lam3 (*SphI*) and lam6 (*HindIII*) listed in Table 1. The 513 bp PCR product was digested by *SphI* and *HindIII* restriction enzymes and cloned into the pQE30 vector (Qiagen) containing a 6xHistidine (6xHis) tag just upstream of the multiple cloning site. Expression and purification were done following the QIAexpressionist protocol (Qiagen) under denaturing conditions. The purified agrin fragment of 25kD was analyzed by SDS-polyacrylamide gel electrophoresis (SDS PAGE) and stained with Coomassie blue (GelCode, Pierce). Fractions of the elution peak were pooled and dialysed against PBS^−^ for further experiments.

The agrin fragment encoding both laminin G domains and the C terminus of the protein, was amplified by specific primers, named overlap5′ and lam8euk4 (Table 1). The amplified agrin fragment of 1203 bp was fused to an N-terminal fragment of chicken tenascin-C containing a signal sequence and the epitope for the monoclonal anti-tenascin-C (TNC) antibody anti-Tn60 [100]. The TNC fragment was amplified from pCTN 230 [101] with T3 as the upstream primer and an antisense primer of which the 3′ half was homologous to the template sequence and the 5′ part reverse complementary to the beginning of the agrin fragment (primer agr/TN antisense, Table 1). The two fragments were fused by PCR using the T3 and lam8euk4 primers. The resulting fusion product of 2100 bp was digested with restriction enzymes *Cla I* and *Not I* and cloned into pKSII. Insertion was verified by sequencing and the fragment cloned into the *Kpn I* and *Not I* restriction sites of the expression vector pCEP for expression in HEK293EBNA cells. The cells were transfected with the Fugene™6 reagent (Roche) following the supplier's protocol. Since the recombinant agrin fragment contained the signal peptide of vertebrate TNC, the protein product was secreted into the conditioned medium which was then tested by western blotting with anti-Tn60.

For large scale protein expression the cells were grown in 15cm tissue culture dishes in DMEM/10%FCS until they reached 75% confluency. Then the cells were washed with DMEM without FCS and 24 to 48 hours after the medium change the conditioned medium was collected. Agrin in the conditioned medium was precipitated with 50% ammonium sulphate at 4°C. The pellet was resuspended in PBS^−^/0.1% Tween and dialysed overnight against the same buffer. Agrin was purified over an anti-Tn60 affinity column as described previously for recombinant TNCs [101]. Elution fractions were analysed by SDS-PAGE and western blotting. The fractions containing peak amounts of agrin fragment were pooled and dialysed against PBS^−^. Alternatively, precipitated conditioned medium was dialysed against PBS^−^ overnight and DMEM for one hour to be used for *in vitro* binding assays with dystroglycan (see below).

### Monoclonal and polyclonal antibodies against C. elegans agrin

The recombinant agrin fragment expressed in *E. coli* was used as antigen for the immunization of mice to raise monoclonal antibodies. Conditioned media from hybridoma clones were tested for antibody production and specificity. The activity was determined by ELISA tests and western blots using recombinant agrin protein encompassing both lamG domains expressed in eukaryotic cells. Four hybridoma clones were positive, their cultures were expanded, and the antibodies from conditioned media concentrated over protein G columns. In further experiments a pool of the purified monoclonal antibodies was used.

Polyclonal antibodies were raised in two rabbits (AG1 and AG2) against the recombinant agrin fragment containing both lamG domains fused to a short epitope of TNC. Antisera were tested by western blot and immunofluorescence for the specificity to the agrin fragment and preimmune sera, taken from the rabbits just before the immunization, served as negative control. To purify monospecific antibodies from these antisera the recombinant agrin fragment used as antigen was bound to CNBr-activated Sepharose™ 4B resin (Amersham Pharmacia) and packed into a column. Polyclonal antiserum from rabbit AG1 was loaded, the column was washed, bound antibodies eluted and their activity tested by western blot of the recombinant protein. These highly purified antibodies were used for the detection of endogenous agrin in *C. elegans*.

### Western blots

The purified agrin fragment of two lamG domains was run on 7.5% SDS PAGE [102] and transferred onto Immobilon membrane (Millipore). The membrane was blocked for one hour in 5% non-fat dry milk (Fluka) in TBS/Tween-20 (0.05%) (Fluka) with gentle agitation at room temperature. Incubation with primary antibodies was carried out overnight at 4°C slowly rocking. The anti-agrin purified monoclonal antibody pool was diluted 1∶1000 and whole polyclonal antisera 1∶8000 in blocking solution (5% non-fat dry milk in TBS/Tween-20). The following day membranes were washed over one hour with several changes of TBS/Tween-20, followed by incubation with goat anti-mouse or goat anti-rabbit horseradish peroxidase (HRP)-conjugated secondary antibodies (Cappel, MP Biomedicals), diluted 1∶2000 in 5% milk, during one hour at room temperature. After extensive washing in TBS/Tween over more than one hour, protein on the membrane was visualized by ECL reagent (Amersham Biosciences) and exposed on Kodak Biomax MR film.

To detect endogenous worm agrin by western blotting young larval stages were homogenized in ice-cold RIPA buffer (NaCl 150 mM, TrisHCl pH8 50 mM, NP-40 1%, deoxycholic acid 0.5%, SDS 0.1%, NaF 50mM) containing a cocktail of protease inhibitiors (Complete Mini EDTA-free, Roche). The suspension was centrifuged for 30 minutes at 14000 rpm in a tabletop centrifuge at 4°C. The supernatant was kept and the pellet resuspended in RIPA buffer including 6M urea and centrifuged for additional 30 min at RT. The supernatant was separated and the pellet resuspended in reducing sample buffer (SB^+^) with 6M urea. All fractions were heated for 5 minutes at 95°C and run on a 6% SDS PAGE, followed by western blotting, as described above. Purified polyclonal antibodies (AG1) were diluted 1∶500 in the blocking solution and anti-rabbit secondary antibody 1∶10000. Immunoreactive proteins were visualized by the HRP substrate Super Signal (Pierce) exposed to double-coated ML film (Kodak) for 1 minute.

### Immunofluorescence

COS cells were grown in 35mm tissue culture dishes and transfected with pCEP-Agrin using Fugene 6 reagent (Roche). 24 hours after transfection the cells were rinsed with PBS^+^ and fixed with 4% PFA in PBS^−^ for 15 minutes at room temperature. Cells were permeabilized with 0,1% TritonX-100 (Fluka) in PBS^−^ during 5 minutes at room temperature. After rinsing with PBS^−^, the cells were blocked with 3% goat serum in PBS^−^ during 15 minutes at room temperature. Primary antibodies were diluted 1∶100 in blocking solution and incubated 2 hours at room temperature. After washing in PBS^−^, the secondary antibody was diluted 1∶1000 in blocking solution and incubated on cells for one hour at room temperature in the dark. Goat anti-mouse and goat anti-rabbit FITC antibodies (Alexa 488, Molecular Probes) were used on separate samples of cells. Together with the secondary antibodies, Hoechst dye (Fluka) was added at dilution 1∶1000 for visualization of cell nuclei. Cells were washed with PBS^−^, rinsed with deionized water to remove traces of salt, and mounted with ProLong Gold antifade reagent (Invitrogen). The pictures were obtained on a Zeiss Z1 upright fluorescence micoscope for multi-dimensional acquisition. The same conditions were used for all the samples, 100× magnification, exposure of 500 ms for FITC and 80 ms for Hoechst.

To simultaneously stain for agrin and endogenous β-dystroglycan in COS cells, cells were treated as described above. The anti- agrin polyclonal antiserum was used at 1∶100 dilution and the monoclonal anti- β-dystroglycan antibody 43DAG1/8D5 (Novocastra, kindly provided to us by Prof. Markus Rüegg) at 1∶100 dilution in blocking solution. The β-dystroglycan antibody was visualized by a goat anti-rabbit secondary antibody coupled with a red dye (Alexa Fluor^®^ 594, Molecular Probes) and anti-agrin by green-labelled goat anti-mouse (Alexa Fluor^®^ 488, Molecular Probes).

### Immunofluorescence staining of endogenous worm agrin

Worms were immunostained following a modified Finney&Ruvkun protocol [103]. Mixed stages of worms, grown on NGM plates, were extensively washed in M9 buffer. The last wash was done with deionised water and the worms were quick-frozen on dry ice/ethanol in Ruvkun fixation buffer mix diluted from a 2× stock (160 mM KCl, 40 mM NaCl, 20 mM Na_2_EGTA, 10mM spermidine-HCl, 30mM Pipes, pH 7.4, and 50% methanol) with the addition of 1.5% formaldehyde. Following permeabilization by freeze-thaw in three cycles, worms were fixed for 1 hour on ice. The cuticle reduction and final permeabilisation was done by TTB and 1% β-mercaptoethanol overnight, slowly rotating at 37°C. Permeabilization was completed the following day by 10mM dithiothreitol (DTT) in 1× BO_3_ buffer (diluted from 20× stock: 1M H_3_BO_3_, 0.5M NaOH), including 0.01% Triton and 0.3% H_2_O_2_ in the same buffer for additional 15 minutes. The worms were blocked in AbB buffer (1× PBS, 0.1% BSA, 0.5% TritonX-100, 0.05% Na-azide, and 1mM EDTA) and immunostained with the antibody solution in the AbA buffer (1% BSA) overnight at 4°C with gentle rocking. The mouse monoclonal anti-agrin antibody pool used at 1∶200 dilution, the rabbit polyclonal anti-agrin antibody at 1∶100, and a rabbit polyclonal anti-rim antibody 1∶6000 [69]. The anti-rim was kindly provided by Prof. Michael Nonet. Worms were washed with AbB during several hours and incubated with the secondary antibody at 1∶1000 dilution in AbA buffer at room temperature gently rocking in the dark during two hours. For agrin monoclonal antibodies anti-mouse secondary antibody conjugated with a green dye (Alexa Fluor^®^ 488, Molecular Probes), for polyclonal anti-agrin secondary anti-rabbit labeled green (Alexa Fluor^®^ 488, Molecular Probes) and for rim polyclonal antibody red-labeled anti-rabbit (Alexa Fluor^®^ 546, Molecular Probes) was used. Following extensive washing in AbB during two hours, the worms were mounted on glass slides with Mowiol (Dabco) mounting medium. Images of agrin/rim co-staining were obtained by a confocal LSM510 META Axioplan2 microscope and for single agrin stainings by the polyclonal antibody a Zeiss Axioscope Bio microscope was used.

### In vitro assay for agrin-β-dystroglycan binding

Purified chicken α-dystroglycan ([104]; kind gift from Prof. Markus Rüegg) was run on 7.5% SDS PAGE (2.5 µg protein per lane) and transferred to a nitrocellulose membrane. The membrane was blocked for two hours in blocking buffer (PBS, 0.05% Tween-20, 1mM CaCl_2_, 1mM MgCl_2_, 5% milk). The membrane was cut in strips which were incubated with different samples of agrin. Chicken recombinant agrin proteins were kindly provided by Prof. M. Rüegg [97]. The chicken agrin fragments were 120 kDa in size, containing 25 kDa of N-terminal laminin-binding domain (NtA) fused to all three C-terminal lam G domains of 95 kDa, differing only in the alternative splicing essential for dystroglycan binding [21]. The fragment of a splice variant of chicken agrin enriched in muscles was used as positive control for DG binding, the chicken agrin neural isoform as negative control, and *C. elegans* recombinant fragment containing two lamG domains was tested. Final concentration of both agrin control samples was 4 µg/ml. The incubation was carried out overnight at 4°C with gentle agitation. The following day, membrane strips were washed in blocking buffer (5% milk in TBS/Tween-20). Detection of bound agrin fragments was performed as described for western blotting with minor modifications. The polyclonal anti-chick agrin antibodies were prepared by Dr. Shuo Lin and kindly provided to us by Prof. Markus Rüegg. Incubation with the primary antibodies was done during 3 hours at room temperature. Polyclonal anti-chicken agrin antibody was diluted 1∶2000 in 5% milk blocking solution. The *C. elegans* agrin fragment was detected by anti-Tn60 [100] diluted 1∶1000 in 3% BSA blocking solution. Following washing in 5% milk blocking solution, membrane strips were incubated with HRP-conjugated goat anti-mouse antibody diluted 1∶2000, during one hour at room temperature. After extensive washing in TBS/Tween proteins were visualized by ECL reagent (Amersham Pharmacia) and the membranes exposed to Kodak BioMax MR film.

## References

[1] Godfrey EW, Nitkin RM, Wallace BG, Rubin LL, McMahan UJ (1984). Components of Torpedo electric organ and muscle that cause aggregation of acetylcholine receptors on cultured muscle cells.. J Cell Biol.

[2] Nitkin RM, Smith MA, Magill C, Fallon JR, Yao YM (1987). Identification of agrin, a synaptic organizing protein from Torpedo electric organ.. J Cell Biol.

[3] Magill-Solc C, McMahan UJ (1988). Motor neurons contain agrin-like molecules.. J Cell Biol.

[4] Magill-Solc C, McMahan UJ (1990). Agrin-like molecules in motor neurons.. J Physiol (Paris).

[5] Magill-Solc C, McMahan UJ (1990). Synthesis and transport of agrin-like molecules in motor neurons.. J Exp Biol.

[6] McMahan UJ (1990). The agrin hypothesis.. Cold Spring Harb Symp Quant Biol.

[7] Ruegg MA, Bixby JL (1998). Agrin orchestrates synaptic differentiation at the vertebrate neuromuscular junction.. Trends Neurosci.

[8] Bezakova G, Ruegg MA (2003). New insights into the roles of agrin.. Nat Rev Mol Cell Biol.

[9] Rupp F, Payan DG, Magill-Solc C, Cowan DM, Scheller RH (1991). Structure and expression of a rat agrin.. Neuron.

[10] Tsim KW, Ruegg MA, Escher G, Kroger S, McMahan UJ (1992). cDNA that encodes active agrin.. Neuron.

[11] Denzer AJ, Gesemann M, Schumacher B, Ruegg MA (1995). An amino-terminal extension is required for the secretion of chick agrin and its binding to extracellular matrix.. J Cell Biol.

[12] Smith MA, Magill-Solc C (1992). Isolation and characterization of a cDNA that encodes an agrin homolog in the marine ray.. Molecular and Cellular Neuroscience.

[13] Groffen AJ, Buskens CA, van Kuppevelt TH, Veerkamp JH, Monnens LA (1998). Primary structure and high expression of human agrin in basement membranes of adult lung and kidney.. Eur J Biochem.

[14] Tsen G, Halfter W, Kroger S, Cole GJ (1995). Agrin is a heparan sulfate proteoglycan.. J Biol Chem.

[15] Winzen U, Cole GJ, Halfter W (2003). Agrin is a chimeric proteoglycan with the attachment sites for heparan sulfate/chondroitin sulfate located in two multiple serine-glycine clusters.. J Biol Chem.

[16] Kammerer RA, Schulthess T, Landwehr R, Schumacher B, Lustig A (1999). Interaction of agrin with laminin requires a coiled-coil conformation of the agrin-binding site within the laminin gamma1 chain.. Embo J.

[17] Denzer AJ, Brandenberger R, Gesemann M, Chiquet M, Ruegg MA (1997). Agrin binds to the nerve-muscle basal lamina via laminin.. J Cell Biol.

[18] Neumann FR, Bittcher G, Annies M, Schumacher B, Kroger S (2001). An alternative amino-terminus expressed in the central nervous system converts agrin to a type II transmembrane protein.. Mol Cell Neurosci.

[19] Burgess RW, Dickman DK, Nunez L, Glass DJ, Sanes JR (2002). Mapping sites responsible for interactions of agrin with neurons.. J Neurochem.

[20] Ruegg MA, Tsim KW, Horton SE, Kroger S, Escher G (1992). The agrin gene codes for a family of basal lamina proteins that differ in function and distribution.. Neuron.

[21] Gesemann M, Cavalli V, Denzer AJ, Brancaccio A, Schumacher B (1996). Alternative splicing of agrin alters its binding to heparin, dystroglycan, and the putative agrin receptor.. Neuron.

[22] Ferns MJ, Campanelli JT, Hoch W, Scheller RH, Hall Z (1993). The ability of agrin to cluster AChRs depends on alternative splicing and on cell surface proteoglycans.. Neuron.

[23] Glass DJ, Bowen DC, Stitt TN, Radziejewski C, Bruno J (1996). Agrin acts via a MuSK receptor complex.. Cell.

[24] Gautam M, Noakes PG, Moscoso L, Rupp F, Scheller RH (1996). Defective neuromuscular synaptogenesis in agrin-deficient mutant mice.. Cell.

[25] DeChiara TM, Bowen DC, Valenzuela DM, Simmons MV, Poueymirou WT (1996). The receptor tyrosine kinase MuSK is required for neuromuscular junction formation in vivo.. Cell.

[26] Burgess RW, Nguyen QT, Son YJ, Lichtman JW, Sanes JR (1999). Alternatively spliced isoforms of nerve- and muscle-derived agrin: their roles at the neuromuscular junction.. Neuron.

[27] Luo ZG, Wang Q, Zhou JZ, Wang J, Luo Z (2002). Regulation of AChR clustering by Dishevelled interacting with MuSK and PAK1.. Neuron.

[28] Okada K, Inoue A, Okada M, Murata Y, Kakuta S (2006). The muscle protein Dok-7 is essential for neuromuscular synaptogenesis.. Science.

[29] Cheusova T, Khan MA, Schubert SW, Gavin AC, Buchou T (2006). Casein kinase 2-dependent serine phosphorylation of MuSK regulates acetylcholine receptor aggregation at the neuromuscular junction.. Genes Dev.

[30] Hoch W, Ferns M, Campanelli JT, Hall ZW, Scheller RH (1993). Developmental regulation of highly active alternatively spliced forms of agrin.. Neuron.

[31] Gee SH, Montanaro F, Lindenbaum MH, Carbonetto S (1994). Dystroglycan-alpha, a dystrophin-associated glycoprotein, is a functional agrin receptor.. Cell.

[32] Gesemann M, Brancaccio A, Schumacher B, Ruegg MA (1998). Agrin is a high-affinity binding protein of dystroglycan in non-muscle tissue.. J Biol Chem.

[33] Martin PT (2003). Dystroglycan glycosylation and its role in matrix binding in skeletal muscle.. Glycobiology.

[34] Michele DE, Campbell KP (2003). Dystrophin-glycoprotein complex: post-translational processing and dystroglycan function.. J Biol Chem.

[35] Batchelor CL, Winder SJ (2006). Sparks, signals and shock absorbers: how dystrophin loss causes muscular dystrophy.. Trends Cell Biol.

[36] Michele DE, Barresi R, Kanagawa M, Saito F, Cohn RD (2002). Post-translational disruption of dystroglycan-ligand interactions in congenital muscular dystrophies.. Nature.

[37] Durbeej M, Campbell KP (2002). Muscular dystrophies involving the dystrophin-glycoprotein complex: an overview of current mouse models.. Curr Opin Genet Dev.

[38] Bezakova G, Lomo T (2001). Muscle activity and muscle agrin regulate the organization of cytoskeletal proteins and attached acetylcholine receptor (AchR) aggregates in skeletal muscle fibers.. J Cell Biol.

[39] Khan AA, Bose C, Yam LS, Soloski MJ, Rupp F (2001). Physiological regulation of the immunological synapse by agrin.. Science.

[40] Zhang J, Wang Y, Chu Y, Su L, Gong Y (2006). Agrin is involved in lymphocytes activation that is mediated by alpha-dystroglycan.. Faseb J.

[41] Brenner S (1974). The genetics of Caenorhabditis elegans.. Genetics.

[42] Riddle DL, Blumenthal T, Meyer BJ, Priess JR, Riddle DL, Blumenthal T, Meyer BJ, Priess JR (1997). Introduction to *C. elegans*.. *C elegans II*..

[43] Millet AC, Ewbank JJ (2004). Immunity in Caenorhabditis elegans.. Curr Opin Immunol.

[44] Jorgensen EM, Mango SE (2002). The art and design of genetic screens: caenorhabditis elegans.. Nat Rev Genet.

[45] Hobert O (2005). Specification of the Nervous System.. *Wormbook*..

[46] Hobert O, Bulow H (2003). Development and maintenance of neuronal architecture at the ventral midline of C. elegans.. Curr Opin Neurobiol.

[47] Lewis JA, Wu CH, Berg H, Levine JH (1980). The genetics of levamisole resistance in the nematode Caenorhabditis elegans.. Genetics.

[48] Gally C, Eimer S, Richmond JE, Bessereau JL (2004). A transmembrane protein required for acetylcholine receptor clustering in Caenorhabditis elegans.. Nature.

[49] Koga M, Take-uchi M, Tameishi T, Ohshima Y (1999). Control of DAF-7 TGF-(alpha) expression and neuronal process development by a receptor tyrosine kinase KIN-8 in Caenorhabditis elegans.. Development.

[50] Forrester WC, Dell M, Perens E, Garriga G (1999). A C. elegans Ror receptor tyrosine kinase regulates cell motility and asymmetric cell division.. Nature.

[51] Francis MM, Evans SP, Jensen M, Madsen DM, Mancuso J (2005). The Ror receptor tyrosine kinase CAM-1 is required for ACR-16-mediated synaptic transmission at the C. elegans neuromuscular junction.. Neuron.

[52] Ackley BD, Kang SH, Crew JR, Suh C, Jin Y (2003). The basement membrane components nidogen and type XVIII collagen regulate organization of neuromuscular junctions in Caenorhabditis elegans.. J Neurosci.

[53] Zwaal RR, Broeks A, van Meurs J, Groenen JT, Plasterk RH (1993). Target-selected gene inactivation in Caenorhabditis elegans by using a frozen transposon insertion mutant bank.. Proc Natl Acad Sci U S A.

[54] Hutter H, Vogel BE, Plenefisch JD, Norris CR, Proenca RB (2000). Conservation and novelty in the evolution of cell adhesion and extracellular matrix genes.. Science.

[55] Letunic I, Copley RR, Pils B, Pinkert S, Schultz J (2006). SMART 5: domains in the context of genomes and networks.. Nucleic Acids Res.

[56] Bendtsen JD, Nielsen H, von Heijne G, Brunak S (2004). Improved prediction of signal peptides: SignalP 3.0.. J Mol Biol.

[57] Tsalik EL, Niacaris T, Wenick AS, Pau K, Avery L (2003). LIM homeobox gene-dependent expression of biogenic amine receptors in restricted regions of the C. elegans nervous system.. Dev Biol.

[58] Lee RY, Sawin ER, Chalfie M, Horvitz HR, Avery L (1999). EAT-4, a homolog of a mammalian sodium-dependent inorganic phosphate cotransporter, is necessary for glutamatergic neurotransmission in caenorhabditis elegans.. J Neurosci.

[59] Fleming JT, Squire MD, Barnes TM, Tornoe C, Matsuda K (1997). Caenorhabditis elegans levamisole resistance genes lev-1, unc-29, and unc-38 encode functional nicotinic acetylcholine receptor subunits.. J Neurosci.

[60] Bessou C, Giugia JB, Franks CJ, Holden-Dye L, Segalat L (1998). Mutations in the Caenorhabditis elegans dystrophin-like gene dys-1 lead to hyperactivity and suggest a link with cholinergic transmission.. Neurogenetics.

[61] Gieseler K, Bessou C, Segalat L (1999). Dystrobrevin- and dystrophin-like mutants display similar phenotypes in the nematode Caenorhabditis elegans.. Neurogenetics.

[62] Chen L, Krause M, Draper B, Weintraub H, Fire A (1992). Body-wall muscle formation in Caenorhabditis elegans embryos that lack the MyoD homolog hlh-1.. Science.

[63] Rogalski TM, Gilchrist EJ, Mullen GP, Moerman DG (1995). Mutations in the unc-52 gene responsible for body wall muscle defects in adult Caenorhabditis elegans are located in alternatively spliced exons.. Genetics.

[64] Ackley BD, Crew JR, Elamaa H, Pihlajaniemi T, Kuo CJ (2001). The NC1/endostatin domain of Caenorhabditis elegans type XVIII collagen affects cell migration and axon guidance.. J Cell Biol.

[65] Kang SH, Kramer JM (2000). Nidogen is nonessential and not required for normal type IV collagen localization in Caenorhabditis elegans.. Mol Biol Cell.

[66] Thomas JH, Stern MJ, Horvitz HR (1990). Cell interactions coordinate the development of the C. elegans egg-laying system.. Cell.

[67] Strome S (1986). Fluorescence visualization of the distribution of microfilaments in gonads and early embryos of the nematode Caenorhabditis elegans.. J Cell Biol.

[68] Campagnola PJ, Millard AC, Terasaki M, Hoppe PE, Malone CJ (2002). Three-dimensional high-resolution second-harmonic generation imaging of endogenous structural proteins in biological tissues.. Biophys J.

[69] Koushika SP, Richmond JE, Hadwiger G, Weimer RM, Jorgensen EM (2001). A post-docking role for active zone protein Rim.. Nat Neurosci.

[70] Avery L, Shtonda BB (2003). Food transport in the C. elegans pharynx.. J Exp Biol.

[71] Nguyen M, Alfonso A, Johnson CD, Rand JB (1995). Caenorhabditis elegans mutants resistant to inhibitors of acetylcholinesterase.. Genetics.

[72] Hart AC, Sims S, Kaplan JM (1995). Synaptic code for sensory modalities revealed by C. elegans GLR-1 glutamate receptor.. Nature.

[73] White JG, Southgate E, Thomson JN, S. B (1986). The structure of the nervous system of the nematode C. elegans.. Philos Trans R Soc Lond B Biol Sci.

[74] Kaplan JM, Horvitz HR (1993). A dual mechanosensory and chemosensory neuron in Caenorhabditis elegans.. Proc Natl Acad Sci U S A.

[75] Albertson DG, Thomson JN (1976). The pharynx of Caenorhabditis elegans.. Philos Trans R Soc Lond B Biol Sci.

[76] Mullen GP, Rogalski TM, Bush JA, Gorji PR, Moerman DG (1999). Complex patterns of alternative splicing mediate the spatial and temporal distribution of perlecan/UNC-52 in Caenorhabditis elegans.. Mol Biol Cell.

[77] Schedl T, Riddle DL, Blumenthal T, Meyer BJ, Priess JR (1997). Developmental Genetics of the Germ Line.. *C elegans II*..

[78] Merz DC, Alves G, Kawano T, Zheng H, Culotti JG (2003). UNC-52/perlecan affects gonadal leader cell migrations in C. elegans hermaphrodites through alterations in growth factor signaling.. Dev Biol.

[79] Grewal PK, Hewitt JE (2003). Glycosylation defects: a new mechanism for muscular dystrophy?. Hum Mol Genet.

[80] Johnson RP, Kang SH, Kramer JM (2006). C. elegans dystroglycan DGN-1 functions in epithelia and neurons, but not muscle, and independently of dystrophin.. Development.

[81] Rogalski TM, Williams BD, Mullen GP, Moerman DG (1993). Products of the unc-52 gene in Caenorhabditis elegans are homologous to the core protein of the mammalian basement membrane heparan sulfate proteoglycan.. Genes Dev.

[82] Francis R, Waterston RH (1991). Muscle cell attachment in Caenorhabditis elegans.. J Cell Biol.

[83] Williams BD, Waterston RH (1994). Genes critical for muscle development and function in Caenorhabditis elegans identified through lethal mutations.. J Cell Biol.

[84] Lee M, Cram EJ, Shen B, Schwarzbauer JE (2001). Roles for beta(pat-3) integrins in development and function of Caenorhabditis elegans muscles and gonads.. J Biol Chem.

[85] Brown NH (2000). Cell-cell adhesion via the ECM: integrin genetics in fly and worm.. Matrix Biol.

[86] Kramer JM (2005). Basement membranes.. Wormbook..

[87] Krause M, Epstein HF, Shakes D (1995). Techniques for analyzing transcription and translation.. *Caenorhabditis elegans*: Modern Biological Analysis of an Organism.

[88] Sulston JE, Brenner S (1974). The DNA of Caenorhabditis elegans.. Genetics.

[89] L'Hernault SW, Roberts TM, Epstein HF, Shakes D (1995). Cell Biology of Nematode Sperm.. *Caenorhabditis elegans*: Modern Biological Analysis of an Organism.

[90] Gasteiger E, Gattiker A, Hoogland C, Ivanyi I, Appel RD (2003). ExPASy: The proteomics server for in-depth protein knowledge and analysis.. Nucleic Acids Res.

[91] Pagni M, Ioannidis V, Cerutti L, Zahn-Zabal M, Jongeneel CV (2004). MyHits: a new interactive resource for protein annotation and domain identification.. Nucleic Acids Res.

[92] Clamp M, Cuff J, Searle SM, Barton GJ (2004). The Jalview Java alignment editor.. Bioinformatics.

[93] Rice P, Longden I, Bleasby A (2000). EMBOSS: the European Molecular Biology Open Software Suite.. Trends Genet.

[94] Sambrook J, Fritsch EF, Maniatis T (1989). Molecular cloning: a laboratory manual: Cold Spring Harbor Laboratory Press, New York..

[95] Mello CC, Kramer JM, Stinchcomb D, Ambros V (1991). Efficient gene transfer in C.elegans: extrachromosomal maintenance and integration of transforming sequences.. Embo J.

[96] Bessereau JL, Wright A, Williams DC, Schuske K, Davis MW (2001). Mobilization of a Drosophila transposon in the Caenorhabditis elegans germ line.. Nature.

[97] Gesemann M, Denzer AJ, Ruegg MA (1995). Acetylcholine receptor-aggregating activity of agrin isoforms and mapping of the active site.. J Cell Biol.

[98] Chow DK, Glenn CF, Johnston JL, Goldberg IG, Wolkow CA (2006). Sarcopenia in the Caenorhabditis elegans pharynx correlates with muscle contraction rate over lifespan.. Exp Gerontol.

[99] Hedgecock EM, Culotti JG, Thomson JN, Perkins LA (1985). Axonal guidance mutants of Caenorhabditis elegans identified by filling sensory neurons with fluorescein dyes.. Dev Biol.

[100] Pearson CA, Pearson D, Shibahara S, Hofsteenge J, Chiquet-Ehrismann R (1988). Tenascin: cDNA cloning and induction by TGF-beta.. Embo J.

[101] Fischer D, Chiquet-Ehrismann R, Bernasconi C, Chiquet M (1995). A single heparin binding region within the fibrinogen-like domain is functional in chick tenascin-C.. J Biol Chem.

[102] Laemmli UK (1970). Cleavage of structural proteins during the assembly of the head of bacteriophage T4.. Nature.

[103] Finney M, Ruvkun G (1990). The unc-86 gene product couples cell lineage and cell identity in C. elegans.. Cell.

[104] Scotton P, Bleckmann D, Stebler M, Sciandra F, Brancaccio A (2006). Activation of MuSK and binding to dystroglycan is regulated by alternative mRNA splicing of agrin.. J Biol Chem.

